# Molecular signatures of BRCAness analysis identifies PARP inhibitor Niraparib as a novel targeted therapeutic strategy for soft tissue Sarcomas

**DOI:** 10.7150/thno.45763

**Published:** 2020-07-25

**Authors:** Hongyi Li, Jian Tu, Zhiqiang Zhao, Lijuan Chen, Yueting Qu, Hongbo Li, Hao Yao, Xiaoshuai Wang, Dung-Fang Lee, Jingnan Shen, Lili Wen, Gang Huang, Xianbiao Xie

**Affiliations:** 1 Department of Musculoskeletal Oncology, the First Affiliated Hospital of Sun Yat-sen University, Guangzhou, China.; 2 Guangdong Provincial Key Laboratory of Orthopedics and Traumatology, Guangzhou, China.; 3 OrigiMed, Co., Ltd., Shanghai, China.; 4 Department of Integrative Biology & Pharmacology, McGovern Medical School, University of Texas Health Science Center at Houston.; 5 Department of Anesthesiology, Sun Yat-sen University Cancer Center, State Key Laboratory of Oncology in South China, Collaborative Innovation Center for Cancer Medicine, Guangzhou 510060, P. R. China.

**Keywords:** BRCAness, Genomic characteristic, PARP inhibitor, PDX models, Soft tissue sarcomas

## Abstract

**Background:** Patients with advanced soft tissue sarcomas (STS) have a dismal prognosis with few effective therapeutic options. A defect in the homologous recombination repair (HRR) pathway can accumulate DNA repair errors and gene mutations, which can lead to tumorigenesis. BRCAness describes tumors with an HRR deficiency (HRD) in the absence of a germline BRCA1/2 mutation. However, the characteristics of BRCAness in STS remain largely unknown. Thus, this study aimed to explore the genomic and molecular landscape of BRCAness using whole exome sequencing (WES) in STS, aiming to find a potential target for STS treatment.

**Methods:** WES was performed in 22 STS samples from the First Affiliated Hospital of Sun Yat-sen University to reveal the possible genomic and molecular characteristics. The characteristics were then validated using data of 224 STS samples from The Cancer Genome Atlas (TCGA) database and *in vitro* data. The analysis of the potential biomarker for BRCAness was performed. Targeted drug susceptibility and combination therapy screening of chemotherapeutics for STS were evaluated in STS cell lines, cell-line-derived xenografts (CDX), and patient-derived xenografts (PDX).

**Results:** Compared with 30 somatic mutation signatures of cancers, high cosine-similarity (0.75) was identified for HRD signatures in the 22 STS samples using nonnegative matrix factorization. Single nucleotide polymorphism indicated a low mutation rate of BRCA1/2 in the 22 STS samples (11.76% and 5.88%, respectively). However, copy number variation analyses demonstrated widespread chromosomal instability; furthermore, 54.55% of STS samples (12/22) carried BRCAness traits. Subsequently, similar genomic and molecular characteristics were also detected in the 224 STS samples from TCGA and *in vitro*. Poly (ADP-ribose) polymerases (PARP)-1 could be a promising reflection of HRD and therapeutic response. Furthermore, the level of PAR formation was found to be correlated with PARP-1. Subsequently, STS cell lines were determined to be sensitive to PARP inhibitor (PARPi), niraparib. Moreover, based on the screening test of the five common PARPis and combination test among doxorubicin, ifosfamide, dacarbazine, and temozolomide (TMZ), niraparib and TMZ were the most synergistic in STS cell lines. The synergistic effect and safety of niraparib and TMZ combination were also shown in CDX and PDX.

**Conclusions:** BRCAness might be the common genomic and molecular characteristics of majority of STS cases. PARP-1 and PAR could be potential proper and feasible theranostic biomarkers for assessing HRD in patients. STSs were sensitive to PARPi. Moreover, the combination of niraparib and TMZ showed synergistic effect. Niraparib and TMZ could be a promising targeted therapeutic strategy for patients with STS.

## Introduction

Soft tissue sarcoma (STS) is an aggressive mesenchymal malignant tumor 1. Wide resection and radiotherapy remain the traditional strategies for treating STS, and chemotherapy is administered for advanced STS. Because of the advances in multidisciplinary care, the evaluation and care of patients with STS have been improving for decades 2. However, local recurrence and metastasis have shown to occur in approximately 20% and 30% of cases, respectively, in 5 years 3, 4. As a result of the few effective therapeutic options available, the prognosis is dismal, as reflected by the median survival of 12.8-14.3 months in patients with advanced STS 5.

Homologous recombination repair (HRR) plays a fundamental role in DNA double-strand break (DSB) repair, as HRR can utilize the sister chromatids as the repair template to restore the genomic sequence of the broken DNA ends precisely 6. The BRCA1/2 mutation may lead to HRR deficiency (HRD), leading to an accumulation of unrepaired DNA errors and gene mutations. Increasing genomic instability facilitates cell death or tumorigenesis 7. Consistent with the above findings, women carrying the BRCA1/2 mutation were found to be susceptible to ovarian and breast cancer 8. DNA repair relies on base excision repair (BER) if HRD develops. When poly (ADP-ribose) polymerases inhibitor (PARPi) block BER by targeting PARP1/2, synthetic lethality occurs. As described above, ovarian and breast cancers with germline mutations in BRCA1/2 are sensitive to PARPi 9,10. Thus, HRD opens a novel avenue for targeted therapy for these tumors.

Increasing studies have demonstrated that tumors carrying germline mutations in BRCA1/2 as well as tumors harboring HRR-related gene mutations that result in HRD, such as pancreatic cancer, prostate cancer, and leukemia, are sensitive to PARPi 11-16. HRD is considered a signature characteristic of tumors carrying germline mutations in BRCA1/2. Therefore, BRCAness is defined as a trait in tumors with an HRD caused by the absence of a germline BRCA1/2 mutation 17. Some studies report that over 80% of osteosarcomas and most leiomyosarcomas acquire BRCAness traits, which could be therapeutically exploited 18,19. Further, an *in vitro* study showed that osteosarcoma with genetic signatures of BRCAness were susceptible to the PARPi, talazoparib alone or in combination with temozolomide 20. However, as a result of the diversity of histological types, high degree of genetic heterogeneity, and low incidence of STS, the characteristics of BRCAness and the effect of PARPi in STS remain largely unknown. Hence, based on our previous researches 21, 22, we conducted this study to explore the genomic and molecular landscape of BRCAness using whole exome sequencing (WES) in STS, aiming to find a potential target for treating STS.

## Methods

### Identify the characteristics of BRCAness in soft tissue sarcomas

To reveal the possible genomic and molecular characteristics of STS, analyses were performed in 22 STS tumor samples from the First Affiliated Hospital of Sun Yat-sen University by comparing with their matched normal adjacent tissues using WES. Mutation signatures were calculated by nonnegative matrix factorization (NMF), and the mutation signatures were compared with the 30 cancer mutation signatures 23. Furthermore, annotation was performed in integrative single nucleotide polymorphism (SNP) data to identify the genes that contain SNPs. The significantly mutated genes and BRCAness genes were analyzed and compared 17. We further calculated the length, gain, and loss of copy number variation (CNV) using the GISTIC analysis, and we located the locus of the corresponding chromosome or gene using the loss of heterozygosity (LOH) analyses. The number of CNVs fulfilling the specific criteria of a BRCA-like phenotype (> 15 Mb) was calculated as the BRCAness proportion. HRD score, is an unweighted sum of LOH, telomeric allelic imbalance (TAI) and large-scale state transitions (LST) 18, was calculated for each STS sample. The threshold of HRD scores for considering a tumor to be BRCA deficient was set to 35 according to the Youden index analysis. To better depict the genomic and molecular signatures, Circos plots were created using the mutation data of all the 22 samples.

Subsequently, the genomic and molecular characteristics were validated using data from The Cancer Genome Atlas (TCGA) database. Data, including the clinical records, pathology reports, SNP, and CNV, of STS samples were extracted from TCGA via cBioPortal. First, the constitution of the histopathologic subtypes was analyzed. Then, similar analyses as those of the data of the 22 samples were conducted on the TCGA data. Furthermore, we then assessed the HRD score for its association with overall survival using the Cox regression model. The expression of the PARP1 and BRCAness were grouped as high or low expression using receiver operating characteristic (ROC) curve and Youden index analysis for the best predictive cut-off point to estimate the survival of STS patients.

### Reagents

The PARPi, niraparib (MK4827), rucaparib (AG014699), veliparib (ABT-888), olaparib (AZD2281), talazoparib (BMN-673); the topoisomerase II inhibitor, etoposide (HY-13629); and the alkylating agent, temozolomide (HY-17364), doxorubicin (HY-15142A), ifosfamide (HY-17419) and dacarbazine (HY-B0078) were purchased from MedChemExpress (New Jersey, USA). The anti-PARP1 antibody (ab32071), the anti-poly (ADP-Ribose) polymer [10H] (ab14459), the anti-Rad51 antibody (ab133534), the anti-RPA32 antibody (ab76420), the anti-Ki67 antibody (ab15580), and the goat anti-rabbit IgG H&L (Alexa Fluor® 488) (ab150077) were purchased from Abcam (Cambridge, UK). Anti-phospho-H2AX (S139) was purchased from Affymetrix eBioscience (Massachusetts, USA). Anti-β-actin (13E5) and anti-GAPDH (14C10) were purchased from Cell Signaling Technology (Massachusetts, USA). Drugs and antibodies were used according to the associated protocols.

### Patient-derived samples

Patient-derived xenografts (PDXs) were established through the immediate transfer of fresh tumor tissue from a spindle cell sarcoma patient into BALB/C nude mice. The tumor samples for WES were derived from 17 patients with STS. The tumor tissue and paired adjacent normal tissue for western blot (WB) analysis were derived from three patients with spindle cell sarcoma, rhabdomyosarcoma, and undifferentiated pleomorphic sarcoma, respectively. The 123 surgical specimens for immunohistochemistry (IHC) were collected from the tissue bank of the Department of Musculoskeletal Oncology, the First Affiliated Hospital of Sun Yat-sen University. All patients provided written consent under the institutional review board-approved protocols.

### Cell viability

The cytotoxic effects and effects on cell viability following the administration of chemotherapeutic regimens and PARPis were determined on six STS cell lines using MTT [3-(4,5-dimethylthiazol-2-yl)-2,5-diphenyl tetrazolium bromide] (Keygen Bio Tech, China). Briefly, 2000 STS cells were transferred into 96-well plates and incubated for 24 h before the addition of the test compound. The cells were then incubated for 24 h, 48 h, and 72 h at 37 °C with an increasing concentration of regimens, respectively. MTT, at a final concentration of 0.5 mg/ml, was added, and following an incubation of 4 h, formazan crystals were dissolved in dimethyl sulfoxide (DMSO). Cell proliferation curves were constructed by measuring the amount of formazan dye generated by the cellular dehydrogenase activity using a microplate reader (Bio-Tek Instruments, Colmar, France) at a wave length of 490 nm. The concentration of the substance required for 50% growth inhibition (IC_50_) was estimated with GraphPad Prism software (GraphPad Software Inc., San Diego, CA, USA).

### Determination of the combination index

For evaluating the combined effect of niraparib and chemotherapeutic regimens (doxorubicin, ifosfamide, dacarbazine, and temozolomide), six preincubated STS cell lines were cotreated with various concentrations of niraparib alone, chemotherapeutic regimens alone, or the combination of niraparib and chemotherapeutic regimens for 48 h. The cell viability of STS cell lines after the different treatments were first determined using the MTT assay. Then, the data were analyzed with a constant ratio combination design based on the protocol described by CompuSyn software (New York, USA). Bar charts and the normalized isobologram for combo were created to depict the treatment combination index (CI), which followed the median effect principle: CIs < 1, 1, and > 1 indicated synergistic, additive, and antagonistic effects, respectively.

### Cell cycle and apoptosis analysis

The cell cycle and apoptosis were studied using the cell cycle assay and cell apoptosis assay (Keygen, Bio Tech, Jiangsu, China) with FACS flow cytometry software (Beckman, California, USA). Flow cytometry data were analyzed with FlowJo software (TreeStar, USA).

### Western blot analysis

Whole cell protein extracts were detected using the following antibodies as previously described: anti-PARP1 (ab32071, 1:10000), anti-Rad51 (ab133534, 1:2000), anti-phospho-H2AX (S139, 1:1000), anti-β-actin (13E5, 1:2000), the anti-poly (ADP-Ribose) polymer [10H] (ab14459, 1:500) and anti-GAPDH (14C10, 1:2000). Membranes were exposed in a chemiluminescence imaging system (Bio-Rad) following the manufacturer's instructions. WB data were quantified using Image Lab (Bio-Rad), and the intensity of bands were compared using the Image J software (NIH, USA).

### Quantitative real-time polymerase chain reaction (qRT-PCR)

RNA extraction and reverse transcription were performed as described previously 24. Transcript levels were normalized to GAPDH. The following primers were used: GAPDH: forward 5'-GCACCGTCAAGGCTGAGAAC-3' and reverse 3'-AGGTGACCGCAGAAGTGGT-5'; RAD51: forward 5'-CGCTGATGAGTTTGGTGTAGC-3' and reverse 3'-ACGTCTACCTCACCCTCTAC-5'. Gene expression was calculated using the 2 - ΔΔCt method.

### Cell clone formation assay

Tumor cells were digested with 0.25% trypsin/0.02% EDTA solution at the logarithmic phase to obtain a single-cell suspension with culture medium. A total of 500-1,000 cells/well were seeded into the six-well culture plates. After 24 h, the cells were treated for 2 weeks with niraparib alone, TMZ alone, or the combination of niraparib and TMZ. The medium was refreshed every 3 days until the cell clones could be observed with the naked eye. The cells were fixed in 4% paraformaldehyde for 30 min and stained with 1% crystal violet for 1 h. Images were captured with a scanning instrument (Epson, Japan).

### Confocal microscopy

For immunofluorescence (IF) analysis, the cells were cultured on confocal dishes treated with 20 μM of etoposide or 10 μM of niraparib. After a 4 h treatment, IF were performed as described previously 25. The primary antibody was anti-Rad51 antibody and the secondary antibody was conjugated to goat anti-rabbit IgG H&L (Alexa Fluor® 488). Images were captured using a confocal laser microscope (Olympus, Japan) with the 63× objective.

### PARP-1 activity

PARP activity was determined in the cell extracts using the HT Colorimetric PARP/Apoptosis Assay (Trevigen, Maryland, USA) according to the manufacturer's protocol. As per the protocol, 5×10^3^ cells were seeded into a 96-well plate and incubated to one concentration of TMZ and/or niraparib for 48 h. After treatment, the following procedures were performed as previously described 26.

### Transfection of Plasmids and Small-Interfering RNA (siRNA) Molecules

HT-1080 and SK-LMS-1 were transfected with pUC57-PARP1 (0.2 µg/96-well plate, 5 µg/6-well plate) plasmid or siPARP1 (100 nM) (TsingKe Biological Technology, Beijing, China). Transfections were performed using Lipofectamine 3000 Reagent (Invitrogen) following the manufacturer's protocol. qRT-PCR, WB, and MTT were performed 48 h after transfection.

### Cell-derived xenografts

Four-week-old female BALB/C nude mice were purchased from the Model Animal Research Center of Nanjing University (China). Induction of tumor xenografts was performed by subcutaneous injection of 0.1 mL cell suspensions containing 5×10^6^ HT1080 or SK-LMS-1 cells in the logarithmic phase into the right flank of the mice. This study followed the approved Institutional Animal Care and Use Committee (IACUC) protocols and the Guide for the Care and Use of Laboratory Animals. Tumor length (L) and width (W) were measured via electronic calipers twice a week, and the volume was calculated based on the following equation: V = L × W^2^ / 2. When the average tumor size reached 150 mm^3^ for HT1080 and SK-LMS-1, mice were randomly assigned to the vehicle and experimental groups. Mice were treated via oral gavage with vehicles, and the dose of the treatment arms was 100 mg/kg (mpk) niraparib (5 ml/kg in 1% w/v methylcellulose) alone, 60 mg/kg (mpk) TMZ (in 1% w/v methylcellulose) alone, or in combination administered each day for 5 days/week, until the tumors reached an approximate size of 1500 mm^3^, they were harvested.

### Patient-derived xenografts (PDXs)

Tumor specimens were obtained from a patient who underwent surgical resection for right thigh spindle cell sarcoma. Biological material was obtained from patients who signed informed consent, and the animal experiment followed institutional review board-approved protocols (approved by ICE for clinical research and animal trials of the First Affiliated Hospital of Sun Yat-sen University, Guangzhou, China, [2017]189). Freshly resected tumor tissue from a patient was divided into approximately 2×2 mm^3^ fragments for transplantation into 5-week-old female BALB/C nude mice. Single tumor fragments were surgically implanted into the dorsal subcutaneous sites of three anesthetized mice (P1). When P1 tumors reached an approximate size of 1500 mm^3^, they were harvested, fragmented, and re-implanted into additional mice (P2). When sufficient P2 tumor grafts reached a volume greater than 200 mm^3^, the animals were divided into four groups, and treatment was initiated as previously described. When tumors reached an approximate size of 1500 mm^3^, they were harvested.

### Immunohistochemistry (IHC)

IHC detection for *in vivo* tumor samples with antibodies against Ki67, phospho-H2AX, and RAD51 was performed. IHC detection of antibodies against phospho-H2AX, RAD51, and RPA32 was also performed in the surgical specimens of 123 patients with STS. Tissue photographs were obtained with an Olympus CKX41 (400×). The IHC staining scores were determined as described in a previous study 27.

### Statistical analysis

Data were analyzed using IBM SPSS Statistics for Windows, version 19.0 (IBM Corp., Armonk, N.Y., USA). Measurements were analyzed using the two-tailed Student's t test, the Mann-Whitney U test, one-way ANOVA, ROC curve, Youden index, and Pearson or Spearman correlation analysis; categorical data were analyzed with the χ^2^ or Fisher's exact test. Data are presented as the mean± standard deviation (SD), and significant differences are indicated as * *p* < 0.05, ** *p* < 0.01, and *** *p* < 0.001. The analyses of over-all survival (OS) and event-free survival (EFS) were performed using the log-rank test (Mantel-Cox test).

### Ethics approval and consent to participate

Biological material was obtained from patients who signed informed consent, and the animal experiment followed institutional review board-approved protocols, which were approved by the ICE for clinical research and animal trials of the First Affiliated Hospital of Sun Yat-sen University (189/2017).

## Results

### Identifying the characteristics of BRCAness in 22 STS samples

**Figure 1:**
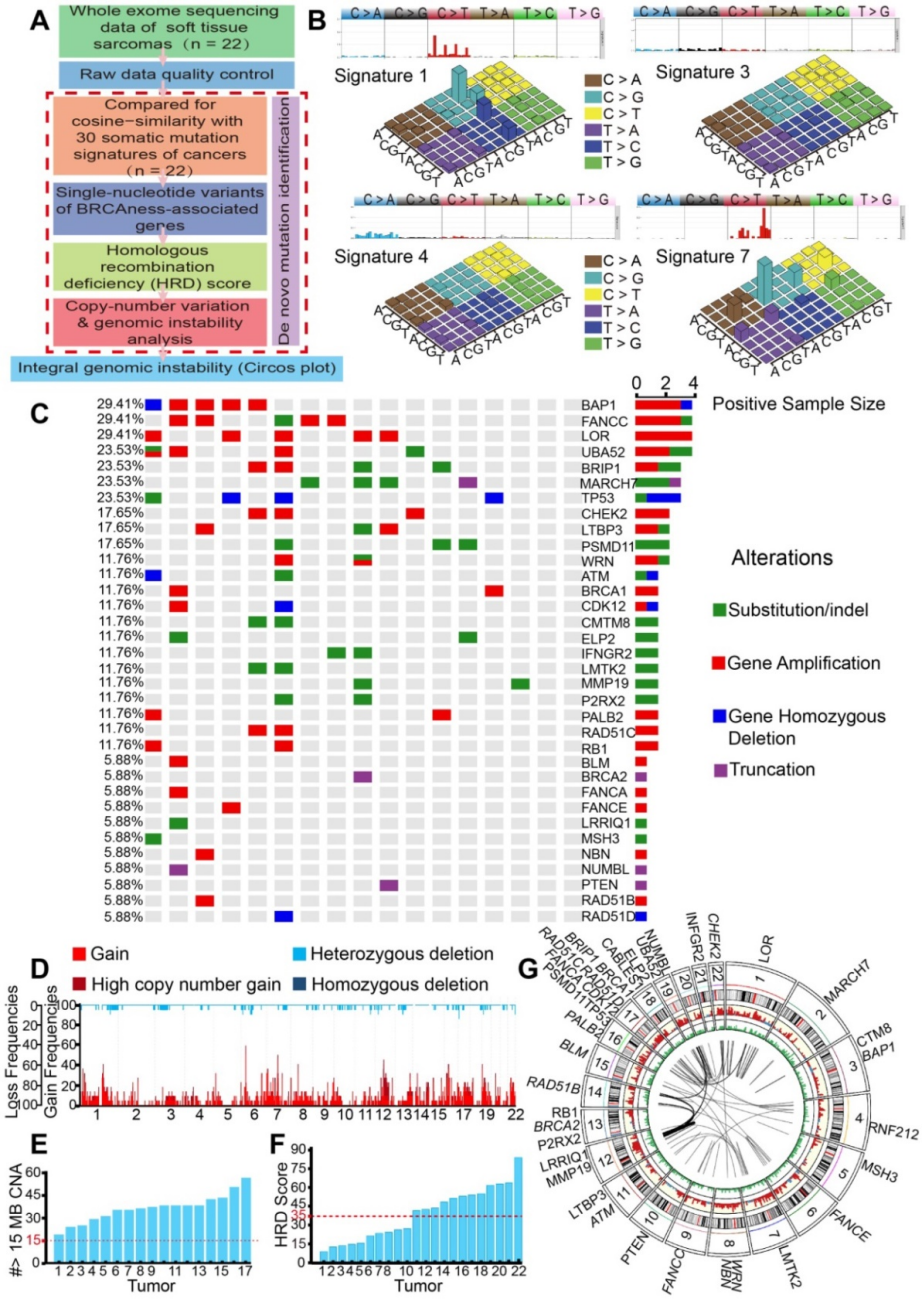
** Genomic and molecular characteristics of BRCAness were observed in 22 STS samples**. **A.** Flow chart of genomic instability analysis from whole exome sequencing (WES) of 22 STS samples from the First Affiliated Hospital of Sun Yat-sen University. **B.** Four signatures out of 30 somatic mutation signatures of cancers were identified in 22 STS samples. The four most similar signatures were signature 1 (cosine-similarity: 0.94, etiology: spontaneous deamination of 5-methylcytosine), signature 3 (cosine-similarity: 0.86, etiology: defects in DNA-DSB repair by homologous recombination), signature 4 (cosine-similarity: 0.76, etiology: smoking), and signature 7 (cosine-similarity: 0.97, etiology: UV exposure). **C.** Distribution of BRCAness-associated gene (BAP1, FANCC, BRIP1, CHEK2, WRN, ATM, BRCA1, ATR, CDK12, PALB2, RAD51C, BLM, BRCA2, FANCA, FANCE, NBN, RAD51B, RAD51D) and the significant mutated gene (LOR, UBA52, MARCH7, TP53, LTBP3, PSMD11, CMTM8, ELP2, IFNGR2, LMTK2, MMP19, P2RX2, RB1, LRRIQ1, MSH3, NUMBL, PTEN) single-nucleotide variants (SNVs) with predicted pathogenic effects and indels across 17 patients with STS. Blue, homozygous deletion; red, amplification; purple, truncation; green, substitution or indel; gray, no alteration. **D.** Copy-number profile of genomes from 22 STS samples. Light red, gain (1 ≤ copy-number variation [CNV] < 2); dark red, high copy-number gain (CNV > 2); wathet, heterozygous deletion (-2 < CNV < -1); navy blue, homozygous deletion (CNV < 2). **E.** The number of CNV meeting the specific criterion of a BRCAness characteristic for each STS tumor (threshold line in red). **F.** Homologous recombination deficiency (HRD) score for each STS tumor (HRD score > 35, threshold line in red). **G.** Circos plot demonstrating the integral mutation in chromosomes, SNVs, CNVs, and gene rearrangements from 22 samples (from outer to inner side); italic font text denotes BRCAness-associated genes.

For the identification of mutation signatures of STS, analyses of the WES data of the 22 samples from 17 STS patients were performed **(Figure 1A)**. The histopathological subtype distribution of the 17 patients included undifferentiated pleomorphic sarcoma (29.41%, n = 5), fibrosarcoma (17.65%, n = 3), synovium sarcoma (11.76%, n = 2), and others **(Figure S1A)**. The detailed clinicopathological characteristics of the 17 patients are shown in **Table S1.**

We then employed NMF for deciphering and comparing the mutation signatures. The four most similar signatures were as follows: signature 7 (cosine-similarity: 0.97, etiology: UV exposure), signature 1 (cosine-similarity: 0.94, etiology: spontaneous deamination of 5-methylcytosine), signature 4 (cosine-similarity: 0.76, etiology: smoking) and signature 3 (cosine-similarity: 0.86, etiology: defects in DNA-DSB repair by HRR). Interestingly, signature 3 was identified in BRCA1/2 mutations or HRD cancers, such as breast, pancreatic and ovarian cancers **(Figure 1B)**. We further analyzed BRCA1/2 alterations in SNP, which affected 5.88% to 11.76% of the 17 patients. We also found high mutation rates in BAP1 (29.41%), FANCC (29.41%), BRIP1 (23.53%), and TP53 (23.53%), which are genes associated with HRD. Universal alterations in the BRCAness-associated genes were detected in further SNP analyses of the 17 patients **(Figure 1C)**. CNV analyses showed overwhelmingly high mutation burden, including gains of the short arm of chromosome 6 in 16 (77.27%), and gains of the long arm of chromosome 7 in 17 (68.18%) cases. Other frequent losses involved 17p (22.73%), 12p (18.18%), 13q (18.18%), etc. Frequent gains involved 6q (63.64%), 7p (63.63%), 7p (68.18%), 2q (59.09%), and LOH could be detected in approximately 22 chromosomes. Together, these data suggested that STS might carry BRCAness integrative genomic characteristics **(Figure 1D)**.

For the further identification of the specific proportion of BRCAness, we calculated the number of CNVs that met the specific criteria of BRCAness (threshold line in red, 15 MB); all the 17 patients with STS contained the characteristics of BRCAness **(Figure 1E)**. We also identified STS as high HRD score tumors, with 54.55% (12/22) of the samples scoring over 35 **(Figure 1F)**. For better depiction of the hallmarks of genomic rearrangement and chromosomal instability, a genome wide Circos plot was created from the integral genomic data and large-scale genome instability could be visualized **(Figure 1G)**.

### BRCAness characteristics were validated in 224 TCGA patients

Given that significant characteristics of BRCAness were found in the 22 STS samples, we performed a validated analysis of 224 STS samples (Sarcoma, TCGA) based on the rich set of TCGA data utilizing the same algorithms. The histopathological subtype distribution of the 224 samples included leiomyosarcoma (40%, n = 90), dedifferentiated liposarcoma (23%, n = 52), undifferentiated pleomorphic sarcoma (19%, n = 43), fibrosarcoma (10%, n = 22) and others **(Figure S1B)**.

**Figure 2:**
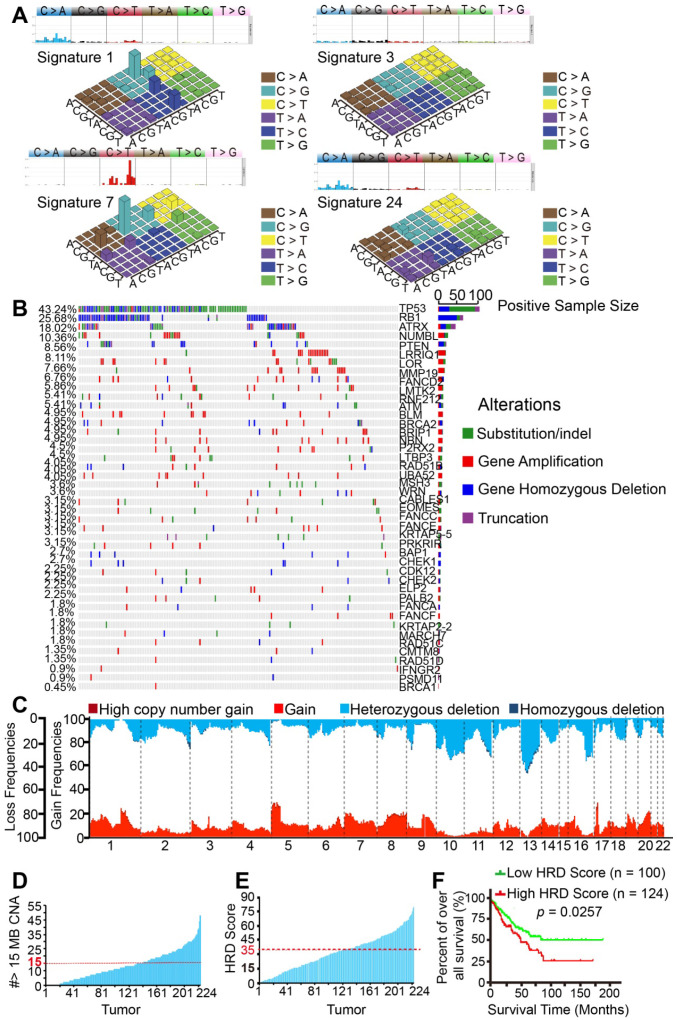
** Genomic and molecular characteristics of BRCAness validated using The Cancer Genome Atlas (TCGA) database. A.** Four signatures out of 30 somatic mutation signatures of cancers were identified in 224 STS samples from TCGA. The four most similar signatures in the TCGA samples were signature 1 (cosine-similarity: 0.92, etiology: spontaneous deamination of 5-methylcytosine), signature 3 (cosine-similarity: 0.87, etiology: defects in DNA-DSB repair by homologous repair), signature 7 (cosine-similarity: 0.97, etiology: UV exposure), and signature 24 (cosine-similarity: 0.57, etiology: exposure to aflatoxin). **B.** Distribution of BRCAness-associated gene (FANCD2, ATM, BLM, BRCA2, BRIP1, NBN, RAD51B, WRN, FANCC, FANCE, BAP1, CHEK1, CDK12, CHEK2, PALB2, FANCA, FANCF, RAD51C, RAD51D, BRCA1) and significant mutated genes single-nucleotide variants with predicted pathogenic effects and indels across 224 STS samples. Blue, homozygous deletion; red, amplification; purple, truncation; green, substitution or indel; gray, no alteration. **C.** Copy-number profile of 224 STS genomes. Light red, gain (1 ≤ copy number variation [CNV] < 2); dark red, high copy-number gain (CNV > 2); wathet, heterozygous deletion (-2 < CNV < -1); navy blue, homozygous deletion (CNV < 2). **D.** The number of CNVs meeting the specific criterion of a BRCAness characteristic for each STS tumor. The threshold for considering a tumor to be BRCA deficient was set to 15 MB CNA (red dashed line). **E.** Receiver operating characteristic (ROC) curve and Youden index analysis were performed to determine the optimal cut-off point for homologous recombination deficiency (HRD) scores in 224 samples from TCGA. According to the ROC curve, the maximal Youden index (sensitivity + specificity - 1 = 0.171) corresponded to HRD scores 34.50; therefore, HRD score 35 was defined to be threshold of BRCAness and could predict the therapeutic response and prognosis of 224 samples from TCGA. The HRD score for each STS tumor (HRD score > 35, threshold line in red). **F.** Comparison of the overall survival rate between the high HRD and low HRD score groups examined using the log-rank test (cutoff: HRD score 35, *p* = 0.0257).

As expected, we found the four most similar signatures in the TCGA samples, which were as follows: signature 7 (cosine-similarity: 0.97, etiology: UV exposure), signature 1 (cosine-similarity: 0.92, etiology: spontaneous deamination of 5-methylcytosine), signature 3 (cosine-similarity: 0.87, etiology: defects in DNA-DSB repair by HRR) and signature 24 (cosine-similarity: 0.57, etiology: treatment with aflatoxin). Interestingly, BRCA1/2 mutants and HRD associated signature 3 was also found in the TCGA samples **(Figure 2A)**. Similarly, BRCA1/2 alterations were detected in 0.45% to 4.95% of SNPs in the 224 samples. We also found high mutation rates of HRD-associated genes TP53 (43.24%), RB1 (25.68%), ATRX (18.02%), and PTEN (8.56%). Widespread alterations in the BRCAness-associated genes were detected **(Figure 2B)**. The most frequent SCNA events were losses of the long arm of chromosome 13 (33.74%). Other frequent losses involved 10q (29.40%), 11q (19.31%), and 16q (25.78%), among others. Frequent gains involved 5p (25.27%), 8q (18.59%), 1p (13.03%), and 1q (12.16%); LOH could be commonly found in approximately 22 chromosomes. Collectively, these data suggested that STS samples from TCGA database might also contain BRCAness integrative genomic characteristics **(Figure 2C)**. Furthermore, 83 (37.05%) of the 224 TCGA samples carried the characteristics of BRCAness **(Figure 2D)**.

To better understand the clinical correlation of HRD, ROC curve and the Youden index (sensitivity + specificity - 1) were performed to determine the optimal cut-off point for HRD scores from TCGA samples. According to ROC, 0.171 was the maximal Youden index corresponding to a HRD score of 34.50. Therefore, a HRD score of 35 was defined as the cutoff threshold of BRCAness. Overall, 44.64% (100/224) of the samples had a HRD score over 35 **(Figure 2E)**. Prognostic analyses were subsequently performed in 224 TCGA patients. Five-year overall survival (OS) analyses demonstrated that patients with high HRD scores (> 35) had a worse prognosis than those with low scores (< 35), implying that HRD scores were associated with adverse outcomes in the 224 samples **(***p* = 0.0257,** Figure 2F)**. Collectively, based on the results of the analyses of our 22 samples and 224 TCGA patients, BRCAness might be the common genomic and molecular characteristics of majority of STS.

### HRD was detected in STS cell lines

**Figure 3:**
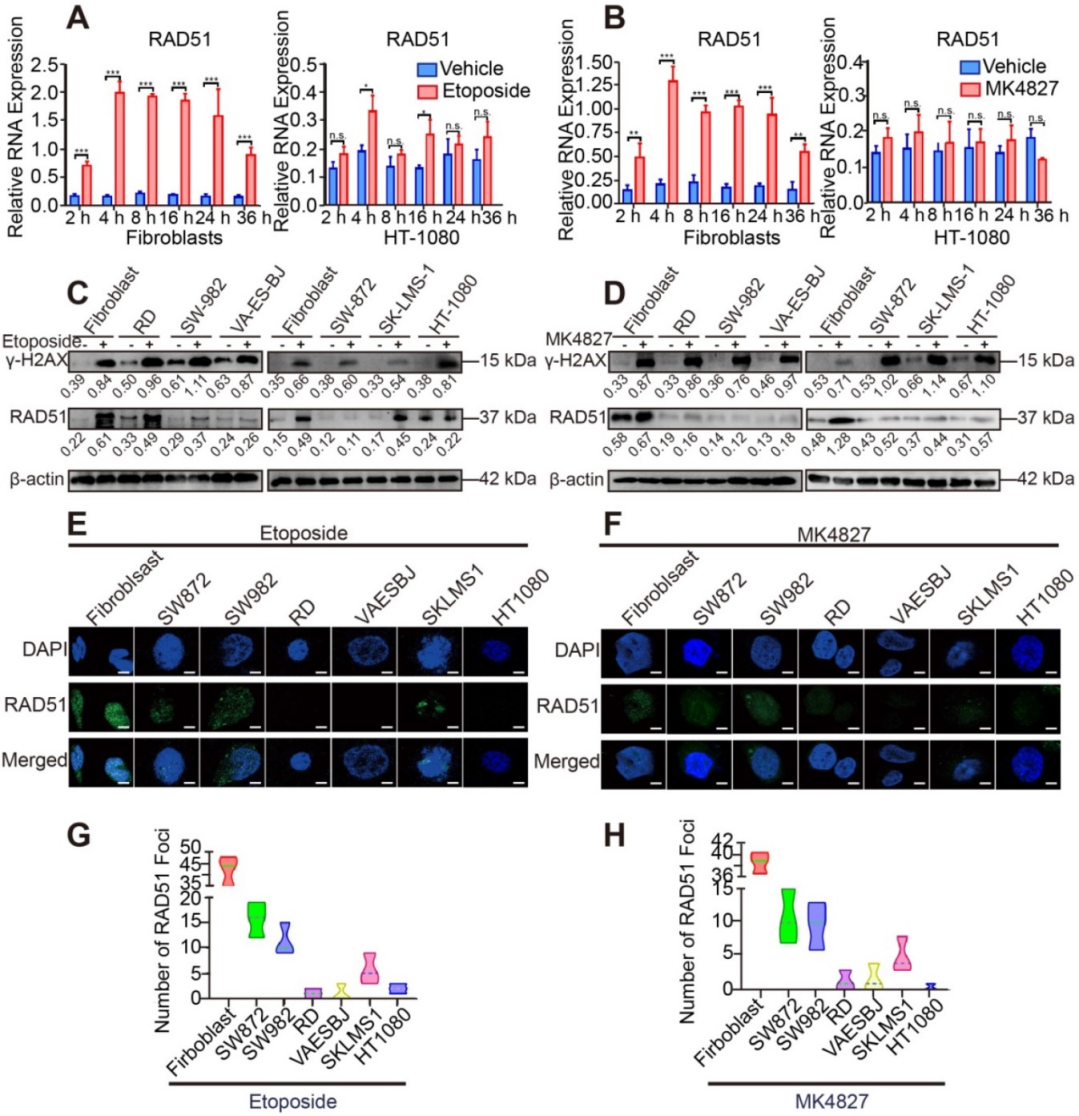
** Impaired DSB repair ability in STS cell lines. A.** Fibroblasts and HT-1080 cells were treated with 20 µM etoposide or DMSO (vehicle group) for 2, 4, 8, 16, 24 and 36 h. qRT-PCR detected that among these timepoints, the relative expression of RAD51 (a marker of homologous recombination repair) was highest at 4 h. **B.** Fibroblasts and HT-1080 cells were treated with 10 µM MK4827 or DMSO (vehicle group) for 2, 4, 8, 16, 24 and 36 h. Again, qRT-PCR detected that the relative expression of RAD51 was highest at 4 h.** C.** When cells were treated with 20 µM etoposide (a topoisomerase II inhibitor) or DMSO (vehicle group) for 4 h, western blots (WB) showed elevated expression of γH2AX, a marker of double-strand breaks (DSBs), in the etoposide group. WB indicated that no significant DSBs developed in the vehicle group, wherein the expression of RAD51 was negligible. However, RAD51 expression was lower in treated STS cells than in treated fibroblasts. **D.** WB demonstrated similar results when STS cell lines were treated with MK4827. **E.** When cells were treated with the same etoposide concentrations described above, an obvious increased green focus of RAD51 was observed in the treated fibroblasts using immunofluorescence (IF) staining. **F.** IF findings were similar when cells were treated with MK4827. **G.** After etoposide treatment, the RAD51 foci in fibroblasts were more than those in STS cell lines. **H.** After MK4827 treatment, the RAD51 foci in fibroblasts were more than those in STS cell lines. HT-1080, fibrosarcoma; SW982, synovial sarcoma; RD, rhabdomyosarcoma; SW872, liposarcoma; SK-LMS-1, leiomyosarcoma; VA-ES-BJ, epithelioid sarcoma; MK4827, niraparib. Scale bar, 5 µm.

To verify the results of the above analyses, we evaluated the activity of HRR in STS cell lines. DNA damage was induced by a topoisomerase II inhibitor (etoposide) and PARPi niraparib (MK4827) in normal fibroblasts and STS cell lines (HT-1080, RD, SW982, VA-ES-BJ, SK-LMS-1, SW872). RAD51, a marker of HRR, showed higher expression in the 4 h treatment group than in the groups with other time points or the control group (DMSO as vehicle) using qRT-PCR **(Figure 3A, B)**. Next, compared with the control group, elevated expression of γH2AX, a marker of DSBs, was detected using WB in the 20 μM etoposide or 10 μM MK4827 treated groups, implying that DSBs had been successfully induced **(Figure 3C, D).** Further, compared with fibroblasts, decreased expression of RAD51 was detected in the six STS cell lines, which suggested that HRR was impaired in the STS cell lines**.** We also conducted an immunofluorescent (IF) assay with the same experimental conditions. We observed a considerable number of RAD51 green foci at the DSB sites in normal fibroblasts that had been treated with etoposide, while few or even no foci were detected in the STS cell lines **(Figure 3E, G)**. A similar phenomenon was found when cells were treated with MK4827 **(Figure 3F, H)**. Consistent with the finding of the previous bioinformatic analyses, STS contained BRCAness traits and often exhibited HRD.

### PARP-1 expression was correlated with HRD and the therapeutic response

**Figure 4:**
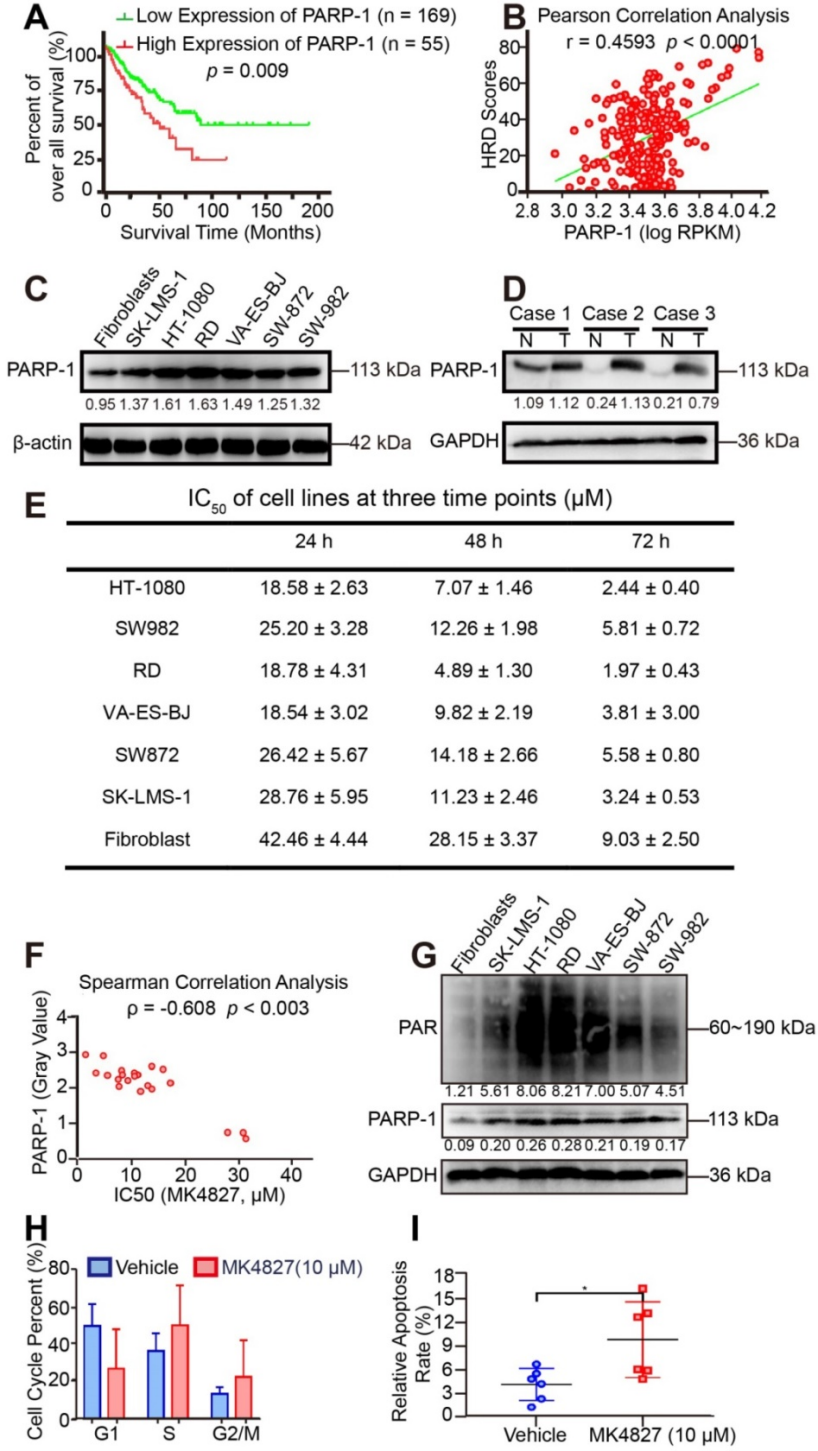
** PARP-1 expression is correlated with HRD and the therapeutic response**. **A.** According to the ROC curve, the maximal Youden index (sensitivity + specificity - 1 = 0.168) corresponded to PARP-1 RPKM 3694.49815; therefore, PARP-1 RPKM 3695 was defined to be the cut-off for the 224 samples. The overall survival rate was compared between the high (n = 55) and low PARP-1 expression (n = 169) groups for the 224 STS patients from TCGA using the log-rank test (cutoff: PARP-1 RPKM 3695, *p* = 0.009). Western blots (WB) indicated higher expression of PARP-1 in six STS cell lines than in fibroblasts. **B.** Pearson correlation analysis showed that the expression level of PARP-1 (RPKM) had a significantly positive correlation with HRD scores in 224 STS cases from the TCGA database (r = 0.4593, *p* < 0.0001). **C.** Western blots (WB) indicated higher expression of PARP-1 in six STS cell lines than in fibroblasts. **D.** WB showed higher expression of PARP-1 in STS tumor tissue than in paired adjacent normal tissue. Case 1, spindle cell sarcoma; case 2, rhabdomyosarcoma; case 3, undifferentiated pleomorphic sarcoma; N, normal tissue; T, tumor tissue. **E.** IC_50_ (µM) of cell lines for three time points (24 h, 48 h, 72 h), denoted as mean ± standard deviation (SD).** F.** Spearman correlation analysis showed that the expression level of PARP-1 (gray value of the protein band on WB) had a moderate negative correlation with the PARPi IC_50_ of cell lines (ρ = -0.608, *p* = 0.003). **G.** WB indicated similar expression patterns between PARP-1 and PAR protein in six STS cell lines and fibroblasts**. H.** Bar chart demonstrating that after 10 µM MK4827 treatment for 24 h, cells arrested in S and the number of cells in G2/M increased. **I.** Plot showing that after 10 µM MK4827 treatment for 48 hours, apoptosis increased. HT-1080, fibrosarcoma; SW982, synovial sarcoma; RD, rhabdomyosarcoma; SW872, liposarcoma; SK-LMS-1, leiomyosarcoma; VA-ES-BJ, epithelioid sarcoma; MK4827, niraparib.

Given that STS, which might carry BRCAness traits, often exhibit HRD. PARPi is the most promising therapy for breast and ovarian cancers with HRD, and PARP-1 is a potential target. As previously reported, PARP-1 plays a fundamental role in single-strand DNA break (SSB) repair, as PARP-1 binds to the damaged DNA at SSBs, which can cause a series of allosteric changes in the structure of PARP-1 that activate its catalytic function 28. This activation leads to the poly ADP-ribosylation (PARylation) and recruitment of DNA repair effectors. To investigate the clinical correlation between PARP-1 and prognosis, prognostic analyses were subsequently performed in 224 TCGA patients. ROC curve and the Youden index were performed to determine the optimal cut-off point for PARP-1 expression level from TCGA samples. According to ROC, 0.168 was the maximal Youden index corresponding to PARP-1 (RPKM) 3694.49815. Therefore, PARP-1 (RPKM) 3695 was defined as the cutoff of low and high expression level. The log-rank test showed that the expression level of the PARP1 was negatively correlated with clinical outcomes in the 224 samples** (***p* = 0.009,** Figure 4A)**. Subsequently, the higher PARP-1 expression was found to be associated with the higher HRD scores (r = 0.4593, *p* < 0.0001, **Figure 4B**). Moreover, WB assays showed increased level of PARP-1 in STS cell lines compared to that in fibroblasts **(Figure 4C)**. Compared with the PARP-1 expression in paired adjacent normal tissue, enriched expression of PARP-1 was also detected in STS tumor tissues of three patients, including high grade spindle cell sarcoma, pleomorphic rhabdomyosarcoma, and high grade pleomorphic undifferentiated sarcoma **(Figure 4D).**

Given that PARP-1 is the direct target of PARPi 29. Besides, the above results suggested that STS might be sensitive to PARPi. Aiming to screen out the ideal PARPi for STS therapy among the five PARPis (talazoparib, niraparib, Olaparib, rucaparib, and veliparib) that have received the US Food and Drug Administration (FDA) approval, the effect of PARPis was assessed using MTT assay in HT-1080. MTT showed that talazoparib had the most potency in HT-1080, followed by niraparib (**Figure S2A, B**). Given that hematologic adverse events are the most common and severe side effects of PARPi, the cytotoxic effect of talazoparib and niraparib was further determined in bone mesenchymal stem cells (BMSC). To further compare the cytotoxic effect of talazoparib and niraparib, the cytotoxic effects at equivalent dose (IC_50_) were determined using MTT assay 30. As **Figure S2C, D** shown, talazoparib at the IC_50_ dose (2 nM) showed a higher cytotoxic activity (30%) in BMSC than did niraparib at its equivalent dose (IC_50_: 7 µM, cytotoxic effect: 10%). Therefore, niraparib was selected for drug treatment assays in this study.

We subsequently investigated the potency of niraparib (MK4827) in STS *in vitro* using MTT assays **(Figure S3A)**. The MTT assay results showed that STS cell lines were sensitive to MK4827. Moreover, with the downregulation of PARP-1 expression, fibroblasts were less sensitive to MK4827 than STS cell lines **(Figure 4E)**. Interestingly, the expression of PARP-1 (gray value of WB of STS cell lines) had a moderate negative correlation with PARPi IC_50_ of cell lines (ρ = -0.608, *p* < 0.003, **Figure 4F**). In other words, the higher PARP-1 expression was associated with higher HRD scores and better therapeutic response of PARPi. Some studies consider the endogenous PARylation as a surrogate marker of PARP activity and functional HRR 31. Our study showed that the level of spontaneous poly (ADP-ribose) (PAR) in STS cell lines and fibroblast control was consistent with PARP-1 (**Figure 4G**). Given that PARP-1 could be a useful predictive biomarker that deserves clinical evaluation. Consequently, PAR also could be a potential predictive biomarker for HRD.

Additionally, we observed increased accumulations in S and G2 phase when STS cell lines had been treated with MK4827 in flow cytometry **(Figure 4H, Figure S3B)**. Moreover, Annexin V/PI flow cytometry detected significant apoptosis induced by MK4827 **(Figure 4I, Figure S3C)**.

### MK4827 and TMZ combination exhibited synergistic effects in STS cell lines

**Figure 5:**
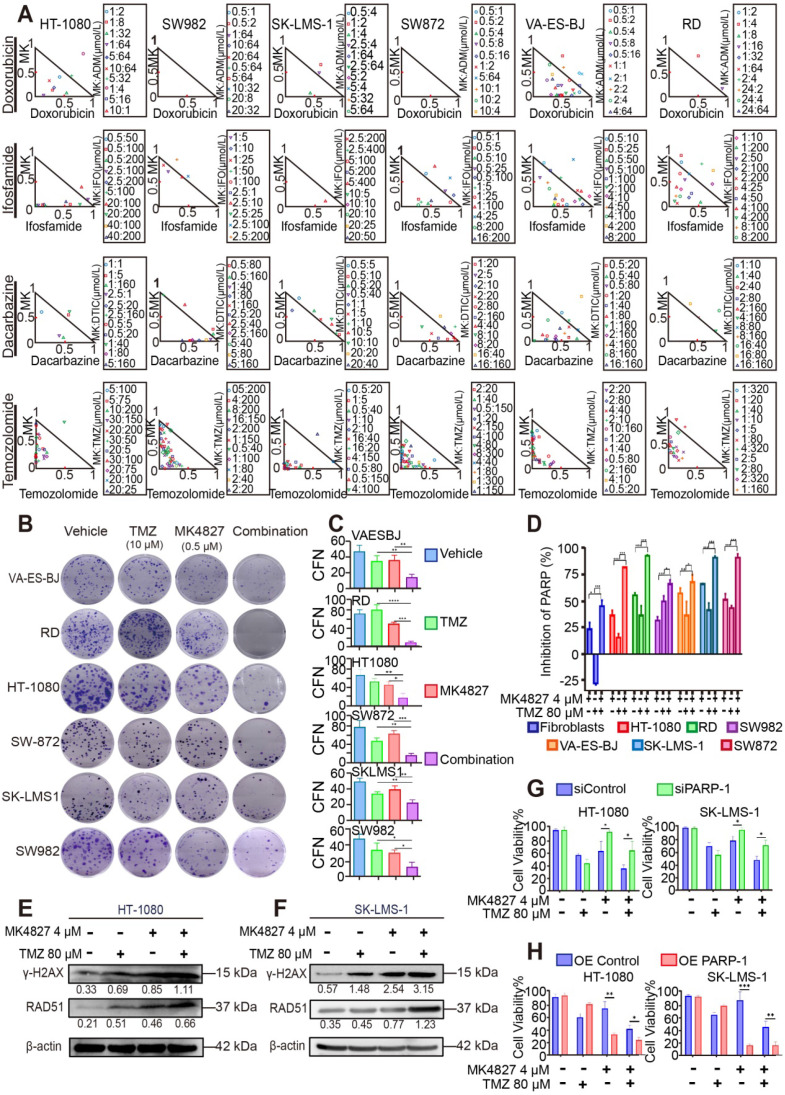
** The investigation for combination effect of MK4827 and temozolomide (TMZ) against STS cell lines *in vitro*. A.** A normalized isobologram for the drug combination was constructed to demonstrate the treatment combination index (CI) of MK4827 and four chemotherapeutic agents for all six cell lines, which follows the median effect principle. CIs < 1, = 1, and > 1 indicate synergistic, additive, and antagonistic effects, respectively. The normalized isobologram showed the synergistic effect of the combination of MK4827 and TMZ against STS cell lines, with CIs < 0.5 for most combinations tested. **B.** Combination therapy had a superior inhibitory effect on colony formation than each reagent alone (treatment for 14 days). **C.** The bar charts reveal the synergistic effects of this combination in inhibiting proliferation, as assessed by colony formation assays. **D.** Measurement of PARP-1 activity during apoptosis indicated that compared with each regimen alone, the MK4827 and TMZ combination augmented DNA damage through a synergistic effect. **E.** Western blots (WB) showed higher expression of γH2AX and RAD51 in HT-1080 cell lines treated with combination therapy than in those treated with MK4827 or TMZ alone. **F.** WB demonstrated higher expression of γH2AX and RAD51 in SK-LMS-1 cell lines treated with combination therapy than in those treated with MK4827 or TMZ alone. **G.** MTT results showing that HT-1080 (left) and SK-LMS-1 (right) were less sensitive to niraparib under PARP-1 knockdown;** H.** MTT results showing that HT-1080 (left) and SK-LMS-1 (right) were more sensitive to niraparib under PARP-1 overexpression**.** CFN: colony formation number; HT-1080, fibrosarcoma; SW982, synovial sarcoma; RD, rhabdomyosarcoma; SW872, liposarcoma; SK-LMS-1, leiomyosarcoma; VA-ES-BJ, epithelioid sarcoma; MK: MK4827 (niraparib); ADM, doxorubicin; IFO, ifosfamide; DTIC, dacarbazine; PARPi: PARP inhibitor; OE: overexpression.

To further screen the synergistic combination therapy for better clinical translation, we tested the combined effect of MK4827 and the common chemotherapeutic reagents for STS treatment, including doxorubicin, ifosfamide, dacarbazine, and temozolomide (TMZ). MTT assays demonstrated that MK4827 and TMZ induced the best synergistic suppressing effect in six STS cell lines compared to the other chemotherapeutic regimens agents **(Figure S4A-D, Figure 5A)**, with the CIs less than 0.5 **(Table S2)**. Consistently, the clone formation assay also demonstrated that MK4827 and TMZ synergistically inhibited the proliferation of STS cell lines** (Figure 5B, C)**.

As described above, TMZ increases SSBs and causes sensitivity to PARPi; thus, we hypothesized that a PARPi and TMZ combination would probably increase the DNA damage. To explore the relationship between DNA damage and the combined activity of niraparib and TMZ, we used the HT Colorimetric PARP/Apoptosis Assay to evaluate the inhibitory effect of MK4827 alone or in combination with TMZ on PARP-1 activity. The assay showed that the MK4827 and TMZ combination induced an increased effect of PARP-1 inhibition compared with either of the single reagents **(Figure 5D)**. To validate the above findings, WB was performed to evaluate the expression of γH2AX and RAD51 when HT-1080 and SK-LMS-1 cells were treated with vehicle, MK4827, TMZ, or in combination, respectively. WB assay detected an elevated expression of γH2AX (marker of DSB) and RAD51 (marker of HRR) when HT-1080 and SK-LMS-1 cells had been treated with a combination of the reagent, compared with that when cells were treated with the vehicle, MK4827, and TMZ, respectively **(Figure 5E, F)**. Overall, these data suggested that MK4827 and TMZ synergistically inhibited the proliferation of STS cell lines by augmenting DNA damage.

To further analyze the role of PARP-1 in the combination effect, the knockdown and overexpression of PARP-1 were subsequently performed in HT-1080 and SK-LMS-1 cells. Efficiency of transduction in HT-1080 and SK-LMS-1 was confirmed by qRT-PCR and WB analysis (**Figure S5A-H**). MTT demonstrated that after PARP-1 knockdown, HT-1080 and SK-LMS-1 were more resistant to PARPi and combination therapy (**Figure 5G**). On the contrary, PARP-1 overexpression resulted in increased sensitivity to the regimens in these cell lines (**Figure 5H**).

### Combining MK4827 and TMZ increased their inhibitory effects *in vivo*

**Figure 6:**
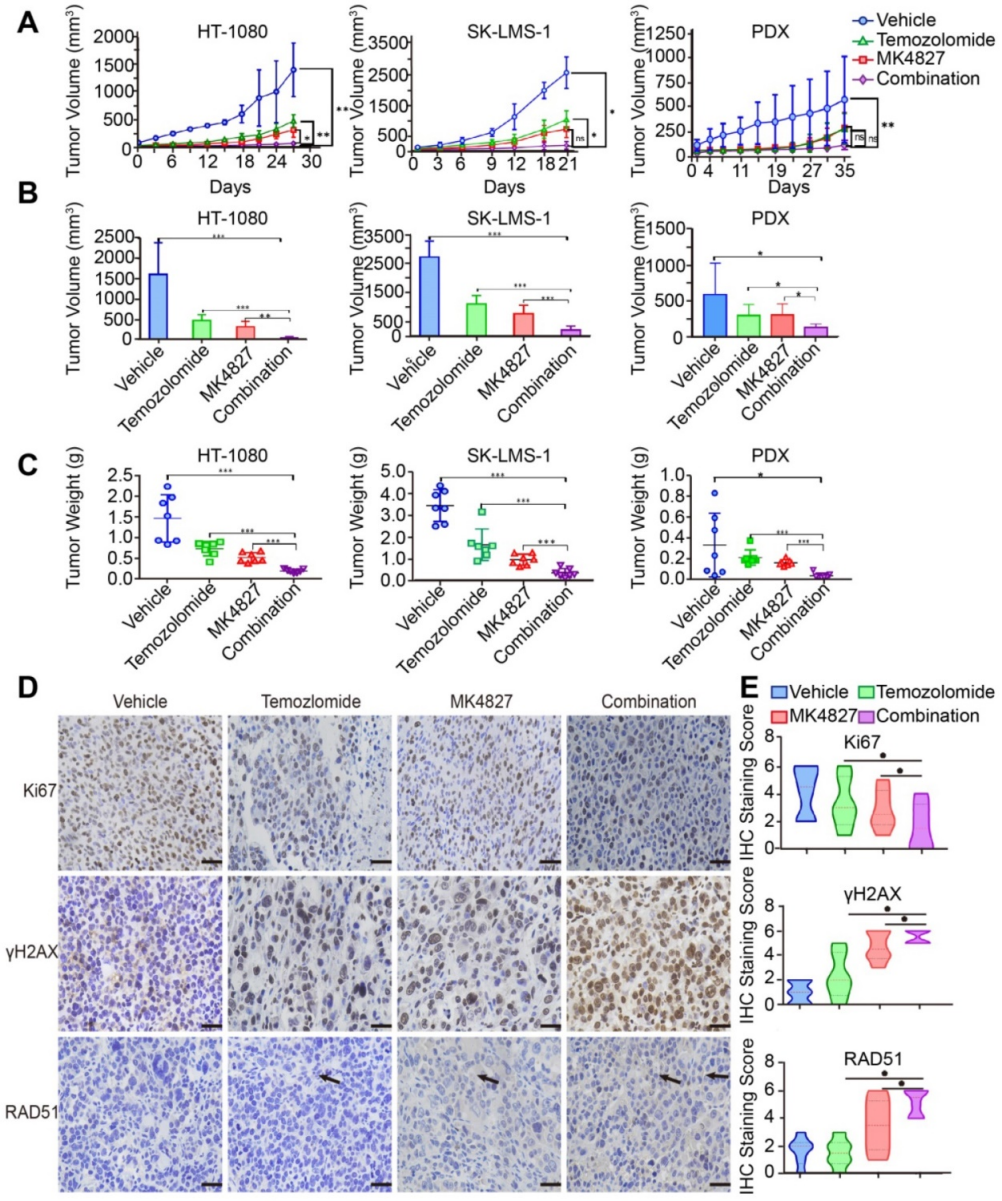
** The combination of MK4827 and temozolomide showed synergistic effects against STS *in vivo*. A.** A subcutaneous tumor model was utilized to assess the effect of the MK4827 and TMZ combination on HT-1080 cells *in vivo*. The tumors were harvested 4 weeks after injection. The tumor volume was measured by electronic calipers twice a week, and the volume was calculated according to the equation V = L × W^2^/2. The growth curve of HT-1080 CDX tumors was shown (left). A subcutaneous tumor model was also used to assess the effect of combination treatment on SK-LMS-1 cells *in vivo*. The tumors were collected at 3 weeks after injection, the growth curve of SK-LMS-1 CDX tumors was shown (middle). A patient-derived xenograft model built using a spindle cell sarcoma was utilized to confirm the synergistic effects. The tumors were collected 5 weeks after surgery. The growth curve of spindle cell sarcoma PDX tumors was shown (right). **B.** Bar charts demonstrated the tumor volumes in the four groups before harvest (HT-1080, n = 7 per group, left; SK-LMS-1, n = 7 per group, middle; PDX, n = 7 per group, right). **C.** The chart displayed the tumor weights before harvest (HT-1080, left; SK-LMS-1, middle; PDX, right). **D.** Representative images of immunohistochemical (IHC) staining against Ki67, γH2AX, and RAD51 in PDX tumor specimens. Scale bar, 100 µm (Positive: Vector Brown). **E.** Plot charts showing the IHC staining scores for Ki67, γH2AX, and RAD51 in PDX tumors. Significant differences were indicated as *: *p* < 0.05, **: *p* < 0.01, ***: *p* < 0.001, and ns: no significance. HT-1080, fibrosarcoma; SK-LMS-1, leiomyosarcoma; MK4827, niraparib.

To confirm the effect of MK4827 and TMZ *in vivo*, we conducted three mouse xenograft model experiments to verify the combined treatment potency *in vivo*. Mice were subcutaneously injected with HT-1080 or SK-LMS-1 cells to develop CDX models. We found that the combination of MK4827 and TMZ could reduce tumor growth in CDX models **(Figure 6A, left and middle)**. To better mimic the tumor heterogeneity, we adopted a spindle cell sarcoma PDX model to further explore the effect of MK4827 and TMZ on STS. A consistent result was observed in the PDX model **(Figure 6A, right)**. The tumor volume and weight in the combination treatment group, measured after sacrificing the mice, were found to be the lowest among the four groups **(Figure 6B, C)**. Together, these results provided supporting evidence that demonstrated an increased inhibitory effect of combining MK4827 and TMZ on STS *in vivo*.

Additionally, we observed that there was no obvious difference in the mice body weight in the control, single reagent, and combination treatment groups during the *in vivo* experiment, which indicated a high safety associated with treatment **(Figure S6A-C)**.

Finally, to further elucidate the mechanisms of cell death *in vivo* model, we performed an assessment of IHC staining for Ki67, γH2AX and RAD51 in the vivo specimens. As a marker for HRR, RAD51-positive cells slightly increased when mice were treated with a combination of the reagents. This phenomenon demonstrated an augmentation of tumor DSBs with a combination of the reagents. We also found that γH2AX was significantly increased in tumors of the combination treatment group compared with tumors of the MK4827 or TMZ treatment groups. In contrast, the expression of Ki67, a cellular marker of proliferation, was markedly reduced in tumors following treatment with MK4827 plus TMZ, compared to treatment with MK4827 or TMZ alone **(Figure 6D, E, Figure S6D, E)**.

### Homologous recombination repair deficiency is a prognostic marker for STS patients

**Figure 7:**
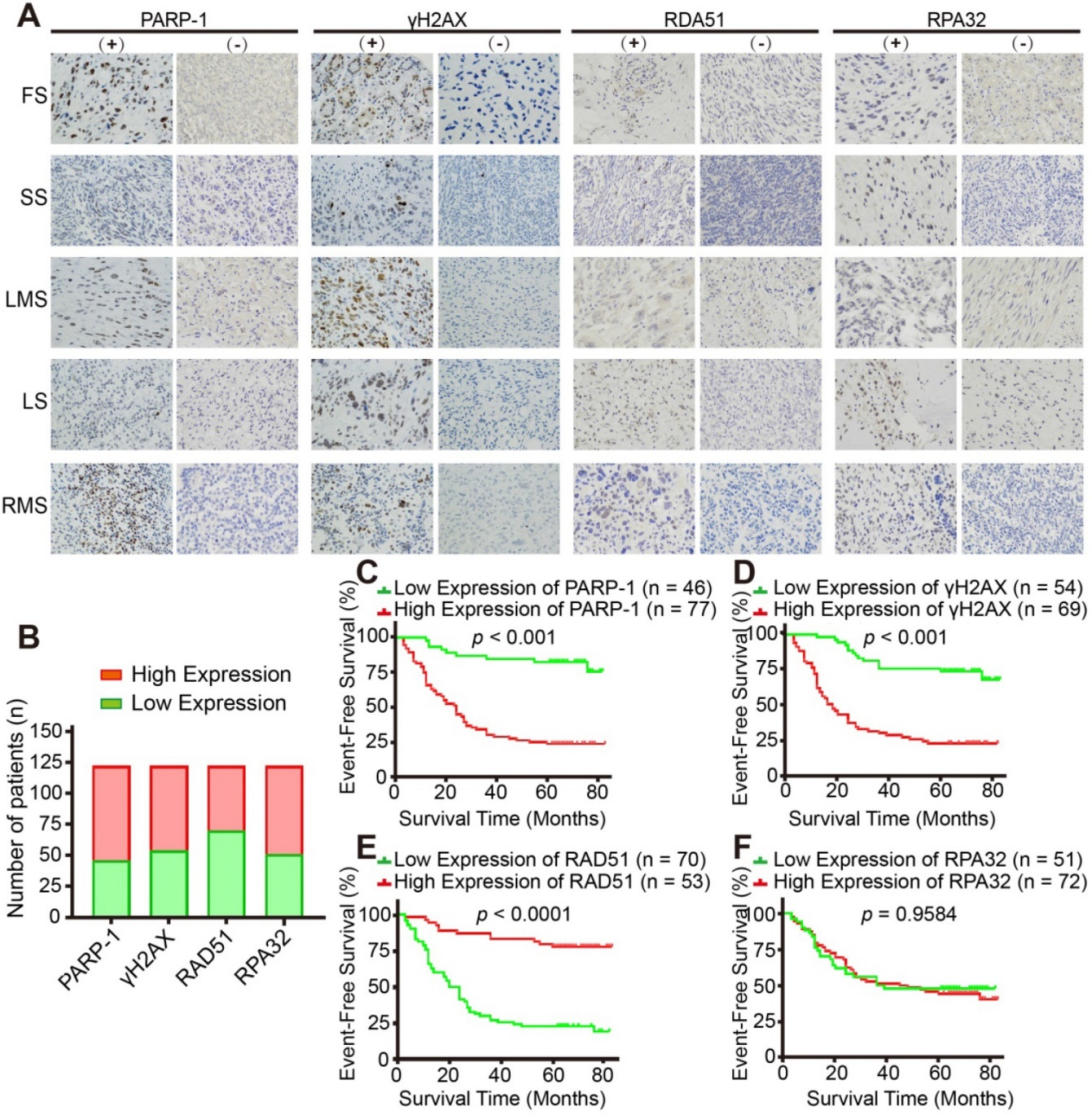
** Expression of HR-associated proteins, including PARP-1, γH2AX and RAD51, significantly correlated with the prognosis of 123 patients with STS**. **A.** Representative images of immunohistochemistry (IHC) staining (positive and negative expression) for PARP-1, γH2AX, RAD51, and RPA32 in samples from patients with fibrosarcoma (FS), synovium sarcoma (SS), leiomyosarcoma (LMS), liposarcoma (LS), and rhabdomyosarcoma (RMS), respectively. (400×, positive: brown). **B.** Bar chart showing the number of patients with positive and negative staining for PARP-1, γH2AX, RAD51, and RPA32 IHC. **C-F.** Log-rank tests showed that the patients in the high RAD51 expression group had better event-free survival (EFS; events were defined as local recurrence, distant metastasis, lung metastasis, and death; *p* < 0.0001), while patients in the high PARP-1 and γH2AX expression groups had worse EFS (*p* < 0.001); RPA32 expression was not associated with the prognosis of the STS patients (*p* = 0.9584). The IHC scores for staining extent were evaluated as follows: 0: 0% of cells showing staining, 1: < 5% of cells showing staining, 2: 5- 50% of cells showing staining, and 3: over 50% of cells showing staining. Staining intensity was scored as 0: negative, 1: weak, 2: intermediate, and 3: strong. The final score was determined as the sum of both the extent and intensity scores. Based on the final score, patients were divided into low (scores 0 and 2) and high (3- 6) expression groups. EFS: event-free survival; FS: fibrosarcoma; SS: synovial sarcoma; LMS: leiomyosarcoma; LS: liposarcoma; RMS: rhabdomyosarcoma.

Finally, we investigated the clinical relevance of HR-associated proteins, including PARP-1, γH2AX, RAD51 and RPA32, in 123 human STS specimens. The major pathologic types of the 123 patients incorporated synovial sarcoma (22%, n = 27), undifferentiated pleomorphic sarcoma (15%, n = 19) and myxoid fibrosarcoma (15%, n = 19, **Figure S1C)**. IHC representative images showed the positive and negative staining of the above-mentioned proteins** (Figure 7A, B)**. Kaplan-Meier analysis indicated that PARP-1 and γH2AX were negatively associated with the event-free survival (EFS) of patients with STS **(***p* < 0.001, **Figure 7C, D)**; in contrast, RAD51 was positively associated with the EFS of patients with STS, which suggested that HRD is a prognostic marker for STS patients **(***p* < 0.0001, **Figure 7E)**. However, no significant relationship between RPA32 and the prognosis of patients was observed (*p* = 0.9584, **Figure 7F)**.

In summary, our study revealed that majority of STS contained the characteristics of BRCAness, and STS demonstrated defects or aberrations in HRR and enrichments in PARP-1. PARP-1 and PAR could be the potential predictive biomarkers for BRCAness. As a putative result, we found that STS cell lines were sensitive to niraparib. Moreover, the combination of niraparib and TMZ exhibited synergistic effects on STS with *in vitro* and *in vivo* experiments. HRD was negatively correlated with the prognosis of patients with STS. This combinational treatment response data suggested that MK4827 and TMZ appear to be a highly promising combination therapy for STS.

## Discussion

The lack of effective regimens has resulted in a constant 50% 5-year overall survival rate and a much lower survival rate for metastatic STS 32. Therefore, there is an urgent need to develop novel treatment strategies for STS. Molecular targeted therapy has been among the most promising remedy, particularly for melanoma, ovarian cancer, and other cancers 8, 33. However, STS appears to be resistant to the current targeted medicine 34. A partial explanation for treatment failure may be because of the different multiple pathological subtypes and several complicated alterations in genes and pathways, of which only a few are targetable 35.

The principal novel findings of our study are as follows: (i) We identified the widespread genomic and molecular characteristics of BRCAness both in our STS samples and in STS samples from the TCGA database, which could be a reliable theoretical basis for PARPi therapy in STS. (ii) The expression level of PARP-1 in STS was a negative prognosis factor and positively correlated with HRD. PARP-1 expression was positively associated with the cytotoxic effect of PARPi. Therefore, PARP-1 could be a promising reflection of BRCAness and therapeutic response. Furthermore, the level of PAR formation was positively correlated with PARP-1, suggesting that PAR could be an appropriate theranostic biomarker of PARPi-based target therapy in STS patients. (iii) Among the five FDA-approved PARPis, niraparib was a more potent and less cytotoxic PARPi for STS treatment. Moreover, according to the screening combination test for cytotoxic regimens therapy for STS (doxorubicin, ifosfamide, dacarbazine, and TMZ), we found that niraparib and TMZ were the most synergistically effective among all STS cell line combination therapies. Our finding provided a novel potential targeted therapeutic strategy for patients with STS.

Knijnenburg *et al.* identified universal DDR gene mutations based on the analyses of 9,125 samples that represented 33 different cancer types from the TCGA PanCanAtlas data. Among 276 mutated DDR genes, HRR was one of the most frequent alterations, and altered HRR function was either positively or negatively correlated with prognosis in different types of cancer 36. In addition, Chudasama *et al.* reported that most leiomyosarcomas demonstrated characteristics of BRCAness, including mutations in HRR-associated DDR genes, multiple structural rearrangements, and abundant specific mutational signatures 19. Our study found widespread HRD both in our STS samples and in STS samples from the TCGA database. Moreover, we identified the genomic instability and HRD was a common molecular characteristic of STS samples.

The identification of biomarkers for evaluating HRD is significant for the application of PARPi in precise treatment. PARP1 is considered to bind to SSB and catalyze the formation of PAR polymers from nicotinamide adenine dinucleotide (NAD**^+^**), and subsequently auto-poly ADP-ribosylation of DNA repair enzymes facilitates their access to SSB lesions 37. Poly (ADP-ribose) polymerase is hyperactivated in tumor cells with HRD 38. Consistent with prior studies, we found that the expression level of PARP-1 was negatively correlated with the prognosis of patients with STS. On the contrary, PARP-1 expression was positively associated with HRD and the cytotoxic effect of PARPi. According to our study, PARP-1 could reflect the BRCAness of STS samples and therapeutic response of PARPi. Previously, PAR formation is reported to be a potential biomarker for HRD 39. Our data revealed that the level of PAR was positively correlated with PARP-1 in six STS cell lines and fibroblasts, which suggested that PAR could be a possible biomarker of PARPi therapy for STS. Our study demonstrated the higher expression level of PARP-1 and PAR formation in STS cell lines and tissues, which provided a reliable theoretical basis for PARPi therapy in STS.

PARPis were first used in clinical trials as a synthetic lethality therapy in BRCA1/2-mutated tumors 40. PARP1 facilitated SSB repair via BER or nucleotide excision repair. PARPi hampered the repair of SSB, which possibly caused the conversion from SSB to DSB. Then, DSB was mainly repaired by HR or NHEJ. Because BRCA1 and BRCA2 are known tumor suppressor genes, cells with BRCA1/2 mutation could lead to HRD. In the absence of a compensatory repair, HRD resulted in the accumulation of DSB, promoted replication fork collapse, and ultimately produced excessive fork collapse, leading to cell death, which was known as synthetic lethality 41. Further studies showed that those BRCA1/2-wide type tumors with HRD remain sensitive to PARPi 42.

As catalytic inhibitors, PARPis could trap the PARP on DNA in cells 43. PARPi can directly interact with the NAD**^+^** binding site for PARP trapping, which lead to a DNA-PARP complex and inhibit DDR. Therefore, the effectiveness and side effects of PARPi depended on the potency of PARPi in trapping PARP onto the DNA 41. So far, PARPis, including talazoparib, niraparib, Olaparib, rucaparib, and veliparib, have received the FDA approval for tumor therapy. The potency of PARPi in trapping PARP was ranked as follows: talazoparib >> niraparib > olaparib = rucaparib >> veliparib 43. Among them, niraparib was the first PARPi that have been approved for ovarian cancer without BRCA1/2 mutation 44. In our study, we found that niraparib had greater potency for STS cells and lower cytotoxicity in normal BMSC than other PARPis. Hence, niraparib was an ideal PARPi for STS therapy.

Combination therapy was deemed as favorable therapeutic strategy for tumors as it had a synergistic response, lower side effect and incidence of drug resistance. Then, we further assessed the combined effect of niraparib and the first-line and the second-line reagents for STS treatment, including doxorubicin, ifosfamide, dacarbazine, and TMZ. Among the four regimens, our study revealed that TMZ in combination with MK4827 induced the best synergistic effect in STS cell lines. Clinically, TMZ is approved as a first-line reagent for pleomorphic glioma and a second-line reagent for advanced STS 45. TMZ gives rise to SSB, which is mainly repaired by BER. Once TMZ causes DNA damage, the damaged bases are recognised by lesion-specific glycosylases. Subsequently an abasic site is generated. In response to DNA damage, PARP-1 binds to SSB lesion and cleaves NAD**^+^** releasing nicotinamide and ADP-ribose. Release of PARylated PARP from the DNA lesion attracts recruitment of essential BER proteins 46. However, PARPi could cause PARP-1 effectively trapped into the lesion, which prevented the access of essential BER proteins to damage sites 47. In that case, PARPi increases the frequency of SSB caused by TMZ 48. Taken together, the combination of niraparib and TMZ therapy is promising in treating patients with STS.

## Conclusions

STS demonstrated BRCAness genomic characteristics. Impaired HRD was observed in STS cell lines. BRCAness could be a common and targetable genomic and molecular characteristic for majority of STS. PARP-1 and PAR could be proper and feasible theranostic biomarkers for assessing HRD in patients with STSs. STSs were sensitive to PARPi, and the combination of niraparib and TMZ was synergistically effective. Collectively, the combination of niraparib and TMZ could be a promising targeted therapeutic strategy for treating patients with STS.

## Supplementary Material

Supplementary figures and tables.

## Abbreviations

BER: base excision repair
BRCA: Breast cancer susceptibility gene
CDX: cell-line-derived xenografts
CNV: copy number variation
DDR: DNA damage repair
DSB: double-strand break
HRD: homologous recombination repair deficiency
HRR: homologous recombination repair
IHC: immunohistochemistry
LOH: loss of heterozygosity
MTT: 3-(4,5-dimethylthiazol-2-yl)-2,5-diphenyl tetrazolium bromide
NHEJ: nonhomologous end joining
NMF: nonnegative matrix factorization
PARP: poly(ADP-ribose) polymerase
PARPi: PARP inhibitors
PDX: patient-derived xenografts
SNP: single nucleotide polymorphism
STS: soft tissue sarcomas
TCGA: the cancer genome atlas
TMZ: temozolomide

## References

[1] Goldblum JR, Folpe AL, Weiss SW, Enzinger FM, Weiss SW Enzinger and Weiss's soft tissue tumors. 6th ed. Philadelphia: Saunders; 2014: 1155.

[2] Clark MA, Fisher C, Judson I, Thomas JM (2005). Soft-tissue sarcomas in adults. N Engl J Med.

[3] Gronchi A, Lo Vullo S, Colombo C, Collini P, Stacchiotti S, Mariani L (2010). Extremity soft tissue sarcoma in a series of patients treated at a single institution: local control directly impacts survival. Ann Surg.

[4] Italiano A, Le Cesne A, Mendiboure J, Blay JY, Piperno-Neumann S, Chevreau C (2014). Prognostic factors and impact of adjuvant treatments on local and metastatic relapse of soft-tissue sarcoma patients in the competing risks setting. Cancer.

[5] Seddon B, Strauss SJ, Whelan J, Leahy M, Woll PJ, Cowie F (2017). Gemcitabine and docetaxel versus doxorubicin as first-line treatment in previously untreated advanced unresectable or metastatic soft-tissue sarcomas (GeDDiS): A randomised controlled phase 3 trial. Lancet Oncol.

[6] Ciccia A, Elledge SJ (2010). The DNA damage response: Making it safe to play with knives. Mol Cell.

[7] Pilié PG, Tang C, Mills GB, Yap TA (2019). State-of-the-art strategies for targeting the DNA damage response in cancer. Nat Rev Clin Oncol.

[8] Rebbeck TR, Mitra N, Wan F, Sinilnikova OM, Healey S, McGuffog L (2015). Association of type and location of BRCA1 and BRCA2 mutations with risk of breast and ovarian cancer. JAMA.

[9] Bryant HE, Schultz N, Thomas HD, Parker KM, Flower D, Lopez E (2005). Specific killing of BRCA2-deficient tumours with inhibitors of poly(ADP-ribose) polymerase. Nature.

[10] Farmer H, McCabe N, Lord CJ, Tutt AN, Johnson DA, Richardson TB (2005). Targeting the DNA repair defect in BRCA mutant cells as a therapeutic strategy. Nature.

[11] Dasa SSK, Diakova G, Suzuki R, Mills AM, Gutknecht MF, Klibanov AL (2018). Plectin-targeted liposomes enhance the therapeutic efficacy of a PARP inhibitor in the treatment of ovarian cancer. Theranostics.

[12] Belz JE, Kumar R, Baldwin P, Ojo NC, Leal AS, Royce DB (2017). Sustained release talazoparib implants for Localized Treatment of BRCA1-deficient Breast Cancer. Theranostics.

[13] Underhill C, Toulmonde M, Bonnefoi H (2011). A review of PARP inhibitors: from bench to bedside. Ann Oncol.

[14] Tuli R, Shiao SL, Nissen N, Tighiouart M, Kim S, Osipov A (2019). A phase 1 study of veliparib, a PARP-1/2 inhibitor, with gemcitabine and radiotherapy in locally advanced pancreatic cancer. EBioMedicine.

[15] Mateo J, Carreira S, Sandhu S, Miranda S, Mossop H, Perez-Lopez R (2015). DNA-repair defects and olaparib in metastatic prostate cancer. N Engl J Med.

[16] Gojo I, Beumer JH, Pratz KW, McDevitt MA, Baer MR, Blackford AL (2017). A phase 1 study of the PARP inhibitor veliparib in combination with temozolomide in acute myeloid leukemia. Clin Cancer Res.

[17] Lord CJ, Ashworth A (2016). BRCAness revisited. Nat Rev Cancer.

[18] Kovac M, Blattmann C, Ribi S, Smida J, Mueller NS, Engert F (2015). Exome sequencing of osteosarcoma reveals mutation signatures reminiscent of BRCA deficiency. Nat Commun.

[19] Chudasama P, Mughal SS, Sanders MA, Hübschmann D, Chung I, Deeg K (2018). Integrative genomic and transcriptomic analysis of leiomyosarcoma. Nat Commun.

[20] Engert F, Kovac M, Baumhoer D, Nathrath M, Fulda S (2017). Osteosarcoma cells with genetic signatures of BRCAness are susceptible to the PARP inhibitor talazoparib alone or in combination with chemotherapeutics. Oncotarget.

[21] Tang QL, Xie XB, Wang J, Chen Q, Han AJ, Zou CY (2012). Glycogen synthase kinase-3β, NF-κB signaling, and tumorigenesis of human osteosarcoma. J Natl Cancer Inst.

[22] Xie XB, Ghadimi MPH, Young ED, Belousov R, Zhu QS, Liu JH (2011). Combining EGFR and mTOR blockade for the treatment of epithelioid sarcoma. Clin Cancer Res.

[23] Alexandrov LB, Zainal SN, Wedge DC, Aparicio SAJR, Biankin SBAV (2013). Signatures of mutational processes in human cancer. Nature.

[24] Meng F, Li Z, Zhang Z, Yang Z, Kang Y, Zhao X (2018). MicroRNA-193b-3p regulates chondrogenesis and chondrocyte metabolism by targeting HDAC3. Theranostics.

[25] Kim MW, Jeong HY, Kang SJ, Jeong IH, Choi MJ, You YM (2019). Anti-EGF receptor aptamer-guided co-delivery of anti-cancer siRNAs and quantum dots for theranostics of triple-negative breast cancer. Theranostics.

[26] Laroche A, Chaire V, Loarer FL, Algéo MP, Rey C, Tran K (2017). Activity of trabectedin and the PARP inhibitor rucaparib in soft-tissue sarcomas. J Hematol Oncol.

[27] Lu JC, Song GH, Tang QL, Yin JQ, Zou CY, Zhao ZQ (2017). MiR-26a inhibits stem cell-like phenotype and tumor growth of osteosarcoma by targeting Jagged1. Oncogene.

[28] Lord CJ, Ashworth A (2017). PARP inhibitors: Synthetic lethality in the clinic. Science.

[29] Heale JT Jr ARB, Schmiesing JA, Kim JS, Kong XD, Zhou S (2006). Condensin I interacts with the PARP-1-XRCC1 complex and functions in DNA single-strand break repair. Mol Cell.

[30] Kalalinia F, Ghasim H, Amel Farzad S, Pishavar E, Ramezani M, Hashemi M (2018). Comparison of the effect of crocin and crocetin, two major compounds extracted from saffron, on osteogenic differentiation of mesenchymal stem cells. Life Sci.

[31] Oplustilova L, Wolanin K, Mistrik M, Korinkova G, Simkova D, Bouchal J (2012). Evaluation of candidate biomarkers to predict cancer cell sensitivity or resistance to PARP-1 inhibitor treatment. Cell Cycle.

[32] Kotilingam D, Lev DC, Lazar AJ, Pollock RE (2006). Staging soft tissue sarcoma: evolution and change. CA Cancer J Clin.

[33] Strub T, Ballotti R, Bertolotto C (2020). The "ART" of epigenetics in melanoma: from histone "alterations, to resistance and therapies". Theranostics.

[34] Linch M, Miah AB, Thway K, Judson IR, Benson C (2014). Systemic treatment of soft-tissue sarcoma-gold standard and novel therapies. Nat Rev Clin Oncol.

[35] Klug LR, Heinrich MC (2017). PDGFRA antibody for soft tissue sarcoma. Cell.

[36] Knijnenburg TA, Wang LH, Zimmermann MT, Chambwe N, Gao GF, Cherniack AD (2018). Genomic and molecular landscape of DNA damage repair deficiency across the cancer genome atlas. Cell Rep.

[37] Satoh MS, Lindahl T (1992). Role of poly (ADP-ribose) formation in DNA repair. Nature.

[38] Gottipati P, Vischioni B, Schultz N, Solomons J, Bryant HE, Djureinovic T (2010). Poly (ADP-ribose) polymerase is hyperactivated in homologous recombination-defective cells. Cancer Res.

[39] Conrad LB, Lin KY, Nandu T, Gibson BA, Lea JS, Kraus WL (2020). ADP-Ribosylation levels and patterns correlate with gene expression and clinical outcomes in ovarian cancers. Mol Cancer Ther.

[40] Fong PC, Boss DS, Yap TA, Tutt A, Wu P, Mergui-Roelvink M (2009). Inhibition of poly(ADP-ribose) polymerase in tumors from BRCA mutation carriers. N Engl J Med.

[41] Liu FW, Tewari KS (2016). New targeted agents in gynecologic cancers: Synthetic lethality, homologous recombination deficiency, and PARP inhibitors. Curr Treat Options Oncol.

[42] Sonnenblick A, de Azambuja E Jr, Azim HA, Piccart M (2015). An update on PARP inhibitors-moving to the adjuvant setting. Nat Rev Clin Oncol.

[43] Pommier Y, O'Connor MJ, de BJ (2016). Laying a trap to kill cancer cells: PARP inhibitors and their mechanisms of action. Sci Transl Med.

[44] Ison G, Howie LJ, Kordestani LA, Zhang LJ, Tang SH, Sridhara R (2018). FDA approval summary: Niraparib for the maintenance treatment of patients with recurrent ovarian cancer in response to platinum-based chemotherapy. Clin Cancer Res.

[45] NCCN. Guideline for soft tissue sarcoma. Version 2. 2020. May 28, 2020.

[46] Zhang J, Stevens MF, Bradshaw TD (2012). Temozolomide: Mechanisms of Action, Repair and Resistance. Curr Mol Pharmacol.

[47] Pommier Y, O'Connor MJ, de BJ (2016). Laying a trap to kill cancer cells: PARP inhibitors and their mechanisms of action. Sci Transl Med.

[48] Van Meir EG, Hadjipanayis CG, Norden AD, Shu HK, Wen PY, Olson JJ (2010). Exciting new advances in neuro-oncology: The avenue to a cure for malignant glioma. CA Cancer J Clin.

