# Supplementary material for: Molecular signatures of BRCAness analysis identifies PARP inhibitor Niraparib as a novel targeted therapeutic strategy for soft tissue Sarcomas
Source: Theranostics. 2020 Jul 25;10(21):9477–94. doi: 10.7150/thno.45763 (PMC7449912; doi:10.7150/thno.45763)
Supplement: Supplementary file 1 — Supplementary figures and tables. [file thnov10p9477s1.pdf]

Supplementary tables and figures

Table S1. Clinicopathological characteristics of 17 patients with soft tissue sarcomas

Patient Number	Age (years)	Gender	Histopathologic Subtype	Location	Enneking Stage
01	59	Male	Undifferentiated pleomorphic sarcoma	Retroperitoneum	III B
02	41	Female	Liposarcoma	Retroperitoneum	I B
03	52	Male	Undifferentiated pleomorphic sarcoma	Thigh	II B
04	24	Male	Synovial sarcoma	Haunch	III B
05	63	Female	Fibrosarcoma	Thigh	II B
06	79	Male	Malignant peripheral nerve sheath tumor	Thigh	III B
07	44	Female	Undifferentiated pleomorphic sarcoma	Thigh	III A
08	27	Male	Synovial sarcoma	Haunch	III B
09	78	Female	Myofibroblastic sarcoma	Knee	II B
10	69	Male	Undifferentiated pleomorphic sarcoma	Thigh	II B
11	25	Female	Alveolar soft part sarcoma	Thigh	II A

12	31	Female	Epithelioid sarcoma	Haunch	III B
13	53	Male	Myofibroblastic sarcoma	Crus	III B
14	50	Male	Undifferentiated pleomorphic sarcoma	Groin	II B
15	30	Female	Fibrosarcoma	Chest wall	II A
16	28	Female	Fibrosarcoma	Forearm	II B
17	64	Male	Malignant peripheral nerve sheath tumor	Waist	II B

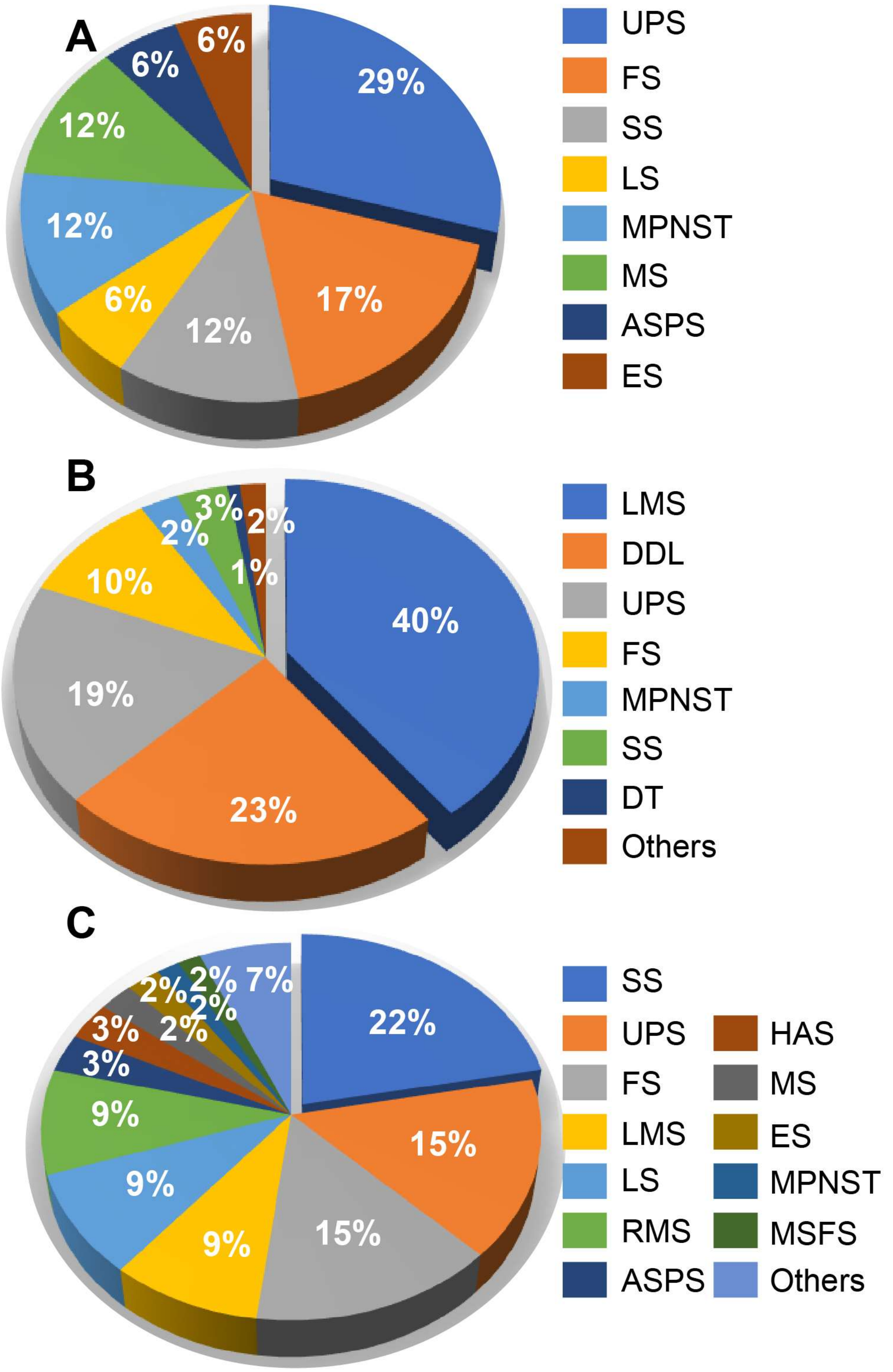

Figure S1. Histopathologic subtypes of the observed patients with STS. A. The distribution of histopathologic subtypes of STS in 17 patients examined using 22 samples subjected to WES analysis**. B**. The distribution of histopathologic subtypes of 224 samples from TCGA subjected to WES analysis. **C**. The distribution of histopathologic subtypes of 123 patients subjected to

immunohistochemical and prognostic analysis. LMS: leiomyosarcoma; DDL: dedifferentiation liposarcoma; UPS: undifferentiated pleomorphic sarcoma; FS: fibrosarcoma; MPNST: malignant peripheral nerve sheath tumor; SS: synovial sarcoma；DT: desmoid tumor; LS: liposarcoma; MS: myofibroblastic sarcoma; ASPS: alveolar soft part sarcoma; ES: epithelioid sarcoma; RMS: rhabdomyosarcoma; HAS: hemangiosarcoma; MSFS: malignant solitary fibrous tumor; WES: whole exome sequencing; TCGA: The Cancer Genome Atlas; STS: soft tissue sarcomas.

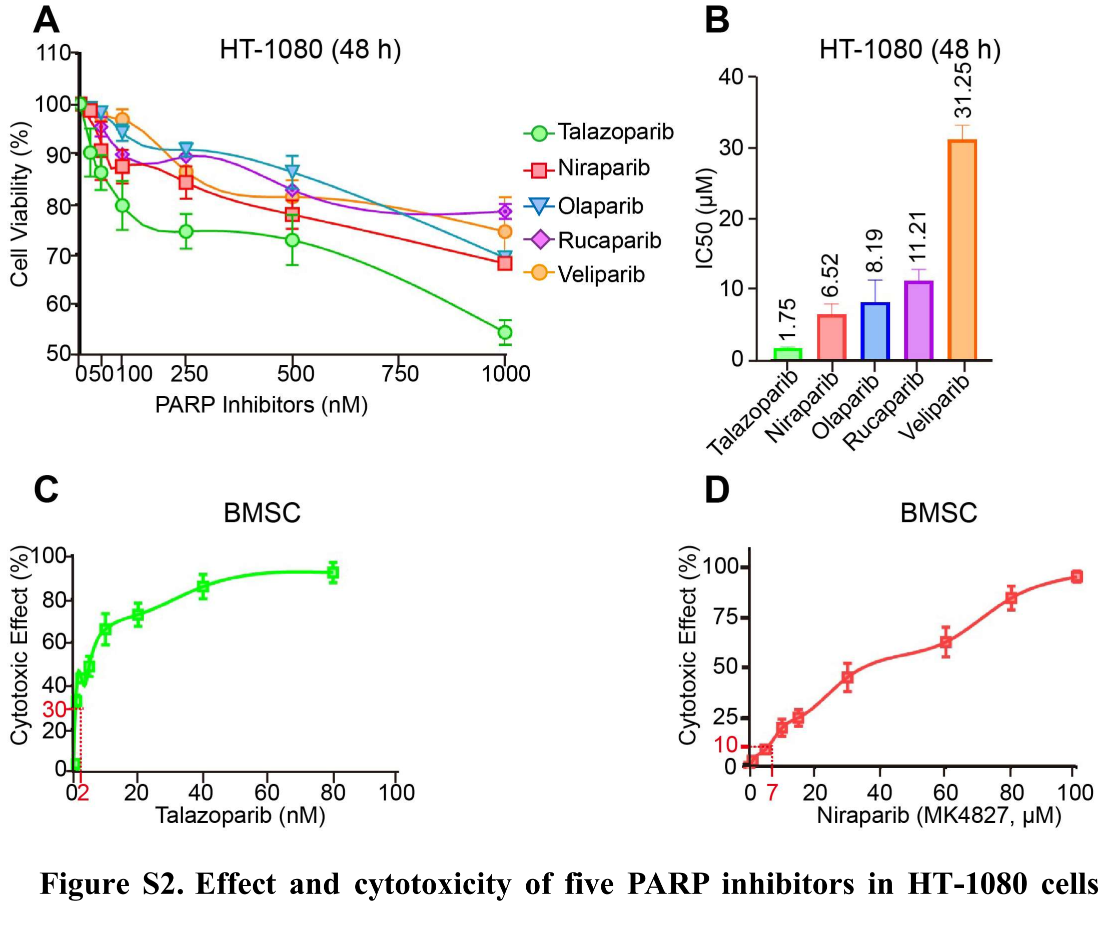

Figure S2. Effect and cytotoxicity of five PARP inhibitors in HT-1080 cells and BMSC. A. MTT assays revealed the sensitivity of HT-1080 cells to different PARPi (48 h). **B.** Bar graph showing 48 h IC_{50} values of five PARPi in HT-1080 cells; the numerical value above the bars represents the mean IC_{50}. **C.** MTT assay demonstrating the cytotoxic effect of talazoparib on BMSC at 48 h; red dashed lines indicate the corresponding cytotoxicity of the IC_{50} dose. **D.** MTT showing

the cytotoxic effect of niraparib on BMSC at 48 h; red dashed lines indicate the corresponding cytotoxicity of the IC_{50} dose. PARPi: PARP inhibitor; BMSC: bone mesenchymal stem cells; MTT: 3-(4,5-dimethylthiazol-2-yl)-2,5-diphenyl tetrazolium bromide.

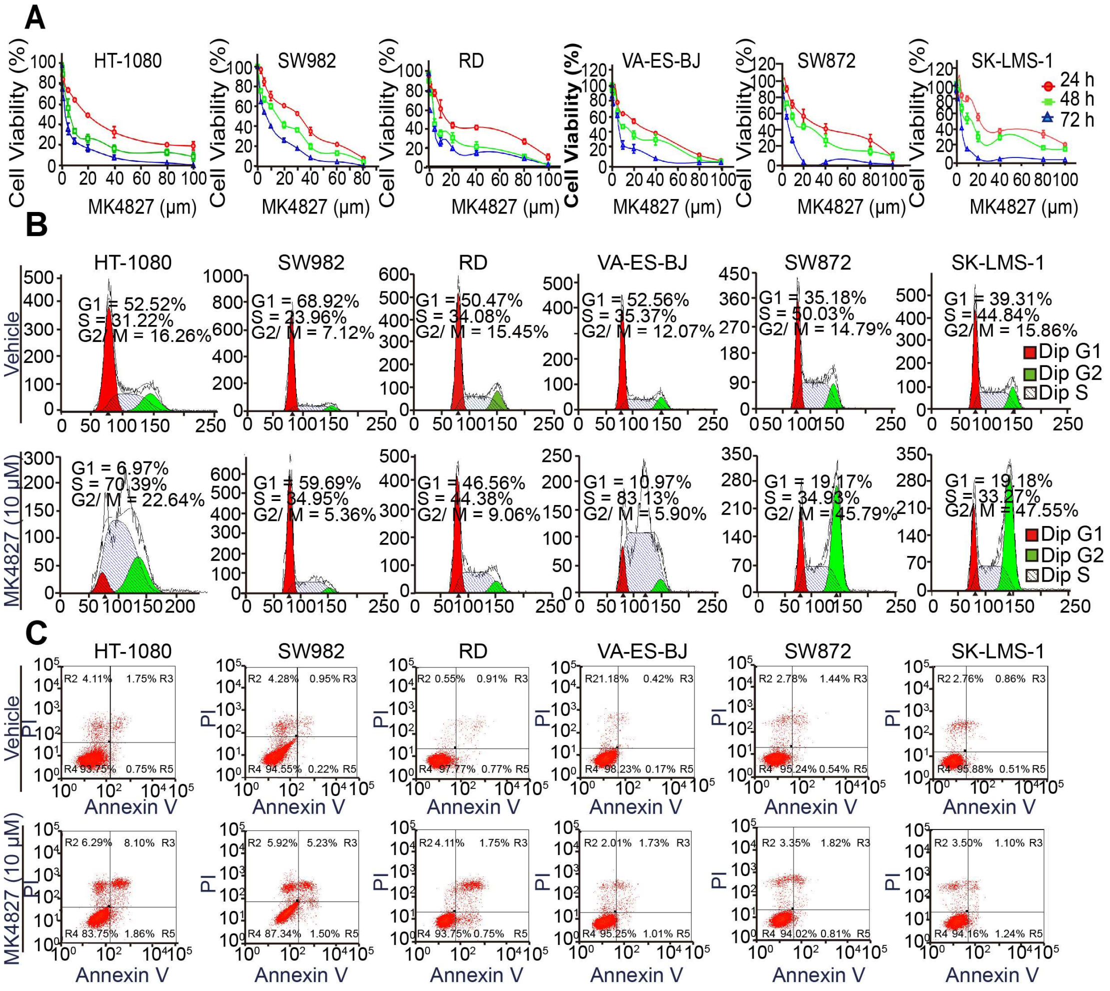

Figure S3. STS cell lines showed sensitivity to niraparib. A. MTT assay demonstrating the dose- and time-dependent sensitivity to niraparib (MK4827) in six STS cell lines. **B.** Flow cytometry indicating that MK4827 induced S and G2/M cell cycle stasis (10 μM MK4827 treatment for 24 h). **C.** Flow cytometry showed that MK4827 increased apoptosis of STS cells (10 μM MK4827 treatment for 48 h). HT-1080, fibrosarcoma; SW982, synovial sarcoma; RD, rhabdomyosarcoma; SW872,

liposarcoma; SK-LMS-1, leiomyosarcoma; VA-ES-BJ, epithelioid sarcoma; MK4827, niraparib.

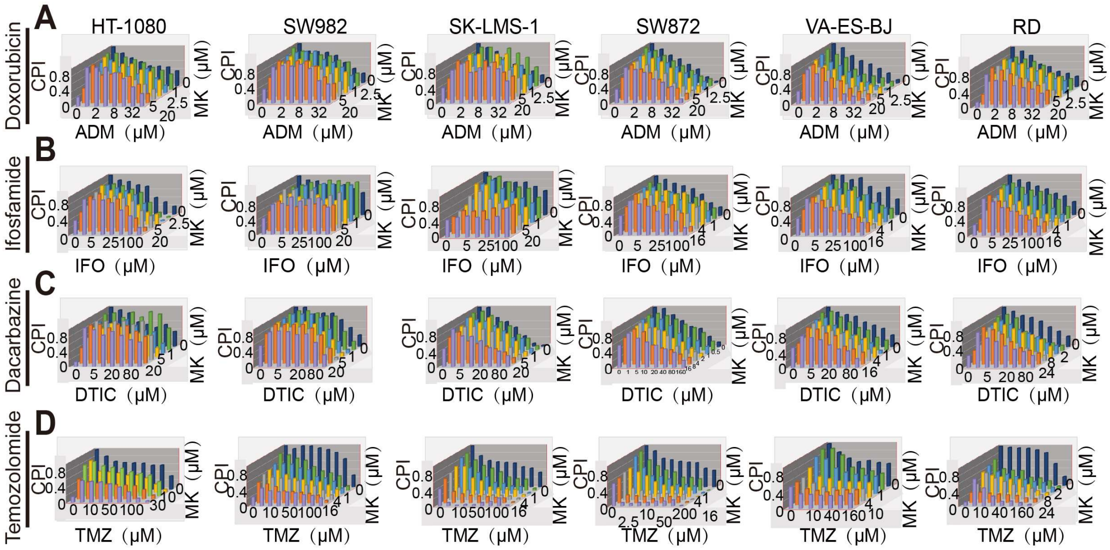

Figure S4. In vitro assays showing the effect of niraparib alone, chemotherapy regimens alone, and a combination of niraparib and chemotherapy regimens on STS. A. 3-D Bar graphs demonstrated the inhibitory effect of MK4827 alone, doxorubicin alone, and a combination of MK4827 with doxorubicin in six STS cell lines. **B.** 3-D Bar graphs showed the combined effect of MK4827 alone, ifosfamide alone, and a combination of MK4827 with ifosfamide in six STS cell lines. **C.** 3-D Bar graphs indicated the potency of MK4827 alone, dacarbazine alone, and a combination of MK4827 with dacarbazine in six STS cell lines. **D.** The combination of MK4827 with temozolomide showed a synergistic effect in six cell lines. CPI: cell proliferation index; ADM, doxorubicin; IFO, ifosfamide; DTIC, dacarbazine; TMZ: temozolomide; STS: soft tissue sarcoma.

Table S2. Combination index and effect assessment in six soft tissue sarcoma cell lines

	Niraparib (MK4827)					
	HT-1080	SW982	SK-LMS-1	SW872	VA-ES-BJ	RD
ADM	1.61 ± 0.92*	2.81 ± 0.83	5.66 ± 3.74	1.95 ± 0.41	0.68 ± 0.17†	2.35 ± 1.02
IFO	1.25 ± 1.01	3.17 ± 1.82	5.44 ± 3.45	1.04 ± 0.36†	0.80 ± 0.25†	0.91 ± 0.48†
DTIC	3.20 ± 3.13	1.26 ± 0.50	1.77 ± 0.69	1.11 ± 0.29	0.72 ± 0.39†	0.86 ± 0.59†
TMZ	0.32 ± 0.19†	0.29 ± 0.09†	0.01 ± 0.01†	0.14 ± 0.10†	0.28 ± 0.05†	0.54 ± 0.31†

*: Mean ± standard deviation (SD).

Combination index (CI) < 1 indicates a synergistic effect, CI = 1 indicates an additive effect, and CI > 1 indicates an antagonistic effect; †: denotes synergistic. ADM, doxorubicin; IFO, ifosfamide; DTIC, dacarbazine; TMZ: temozolomide

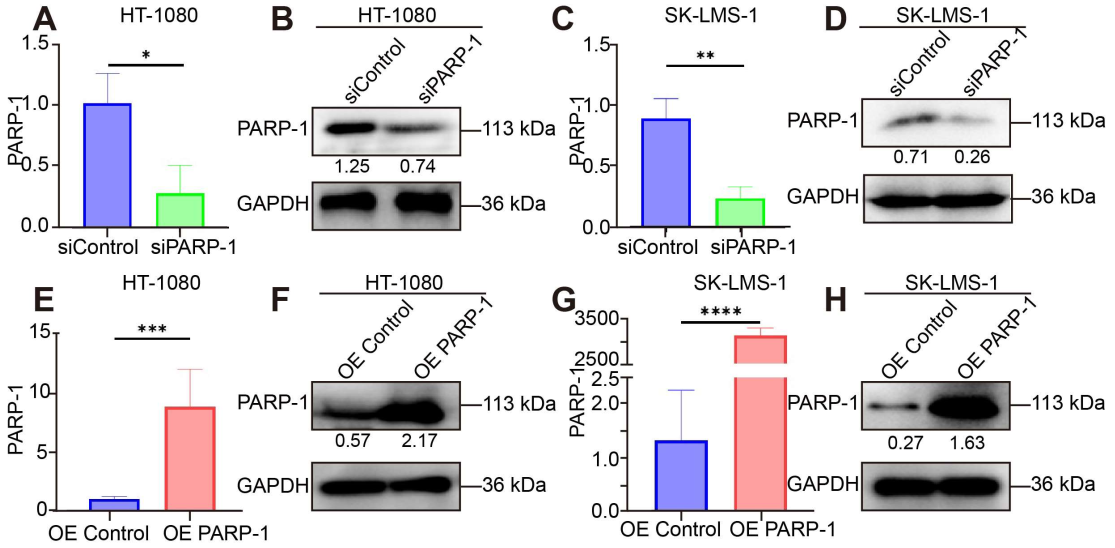

Figure S5. In vitro assays showing the efficiency of PARP-1 knockdown or overexpression in HT-1080 and SK-LMS-1 cells. A. qRT-PCR demonstrating the relative expression of PARP-1 after PARP-1 knockdown in HT-1080 cells. **B.** WB confirming the reduction in PARP-1 protein expression after PARP-1 knockdown in HT-1080 cells. **C.** qRT-PCR demonstrating the relative expression of PARP-1 after PARP-1 knockdown in SK-LMS-1 cells. **D.** WB confirming the reduction in PARP-1 protein expression after PARP-1 knockdown in SK-LMS-1 cells. **E.** qRT-PCR demonstrating the relative expression of PARP-1 after PARP-1 overexpression in HT-1080 cells. **F.** WB confirming the increase in PARP-1 levels after PARP-1 overexpression in HT-1080 cells. **G.** qRT-PCR demonstrating the relative expression of PARP-1 after PARP-1 overexpression in SK-LMS-1 cells. **H.** WB confirming the increase in PARP-1 levels after PARP-1 overexpression in SK-LMS-1 cells. qRT-PCR: quantitative real-time polymerase chain reaction; WB: western blot; OE: overexpression.

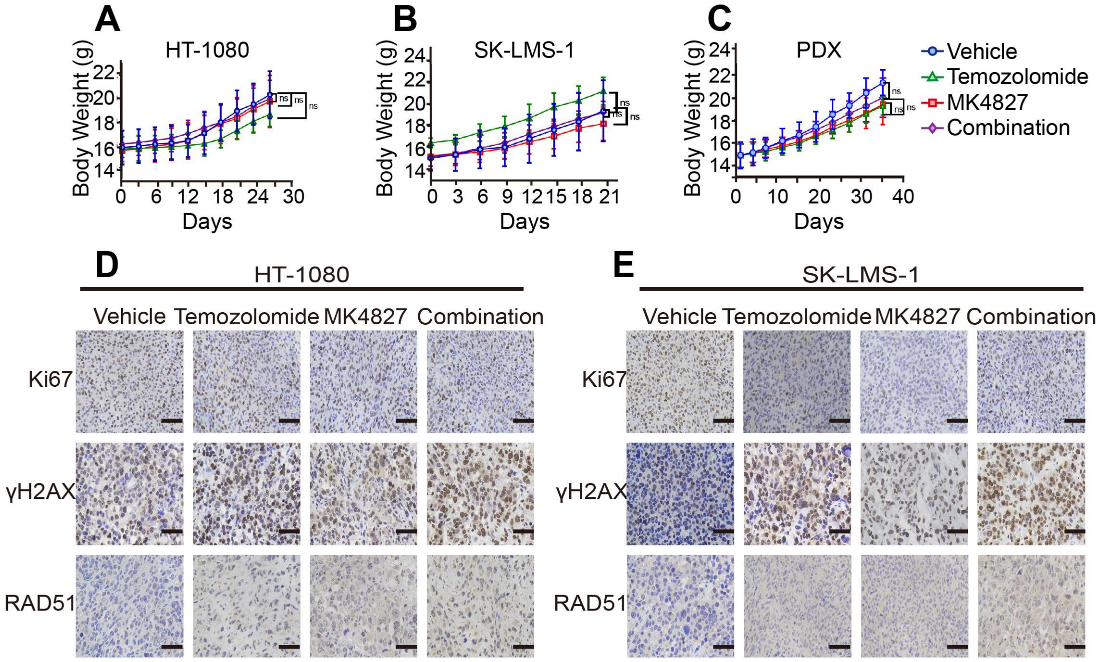

Figure S6. Safety and efficacy of the combination of niraparib and temozolomide in vivo. The growth curve of body weight showed no significant difference among the vehicle, niraparib (MK4827), temozolomide, and combination therapy groups in mice receiving HT-1080 xenografts (**A**), SK-LMS-1 xenografts (**B**), and PDX (**C**). Representative images of immunohistochemical staining for Ki67, γH2AX, and RAD51 in HT-1080 CDX (**D**) and SK-LMS-1 CDX tumors (**E**). (Scale bar, 100 μm; positive: brown). ns: no significance.
